# Faecal volatile organic compounds differ according to inflammatory bowel disease sub‐type, severity, and response to treatment in paediatric patients

**DOI:** 10.1002/ueg2.12603

**Published:** 2024-06-22

**Authors:** Salma Belnour, Rachael Slater, Kukatharmini Tharmaratnam, Marcus Karl‐Heinz Auth, Rafeeq Muhammed, Christine Spray, Duolao Wang, Umer Zeeshan Ijaz, Chris Probert, Stephen Allen

**Affiliations:** ^1^ Faculty of Health and Life Sciences University of Liverpool Liverpool UK; ^2^ Department of Molecular & Clinical Cancer Medicine Institute of Systems, Molecular and Integrative Biology Liverpool UK; ^3^ Department of Health Data Science Institute of Systems, Molecular and Integrative Biology Liverpool UK; ^4^ Paediatric Gastroenterology Alder Hey Children's NHS Foundation Trust Liverpool UK; ^5^ Gastroenterology and Nutrition Birmingham Children's Hospital Birmingham UK; ^6^ Paediatric Gastroenterology Bristol Royal Hospital for Children Bristol UK; ^7^ Department of Clinical Sciences Liverpool School of Tropical Medicine Liverpool UK; ^8^ James Watt South Building University of Glasgow Glasgow UK

**Keywords:** children, Crohn's disease, faecal volatile organic compounds, gas chromatography–mass spectrometry, GC/MS, IBD, ulcerative colitis, VOCs

## Abstract

**Background:**

Faecal volatile organic compounds (VOCs) differ with disease sub‐type and activity in adults with established inflammatory bowel disease (IBD) taking therapy.

**Objective:**

To describe patterns of faecal VOCs in children newly presented with IBD according to disease sub‐type, severity, and response to treatment.

**Methods:**

Children presenting with suspected IBD were recruited from three UK hospitals. Children in whom IBD was diagnosed were matched with a non‐IBD child for age, sex, and recruitment site. Faecal VOCs were characterised by gas chromatography–mass spectrometry at presentation and 3 months later in children with IBD.

**Results:**

In 132 case/control pairs, median (inter‐quartile range) age in IBD was 13.3 years (10.2–14.7) and 38.6% were female. Compared with controls, the mean abundance of 27/62 (43.6%) faecal VOCs was statistically significantly decreased in Crohn's disease (CD), ulcerative colitis (UC) or both especially amongst ketones/diketones, fatty acids, and alcohols (*p* < 0.05). Short‐chain, medium chain, and branched chain fatty acids were markedly reduced in severe colitis (*p* < 0.05). Despite clinical improvement in many children with IBD, the number and abundance of almost all VOCs did not increase following treatment, suggesting persistent dysbiosis. Oct‐1‐en‐3‐ol was increased in CD (*p* = 0.001) and UC (*p* = 0.012) compared with controls and decreased following treatment in UC (*p* = 0.01). In CD, propan‐1‐ol was significantly greater than controls (*p* < 0.001) and extensive colitis (*p* = 0.001) and fell with treatment (*p* = 0.05). Phenol was significantly greater in CD (*p* < 0.001) and fell with treatment in both CD (*p* = 0.02) and UC (*p* = 0.01).

**Conclusion:**

Characterisation of faecal VOCs in an inception cohort of children with IBD reveals patterns associated with diagnosis, disease activity, and extent. Further work should investigate the relationship between VOCs and the microbiome in IBD and their role in diagnosis and disease monitoring.

## Graphical abstract

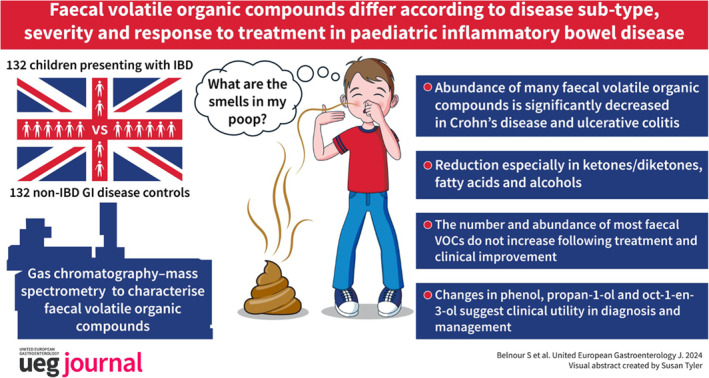

**Key summary**

**Summarize the established knowledge on this subject**

- The pathogenesis of inflammatory bowel disease (IBD) remains poorly understood.
- Investigations for the diagnosis and monitoring of IBD in children can cause distress and delay the start of treatment.
- Measurement of volatile organic compounds (VOCs) in stool is simple and inexpensive.

**What are the significant and/or new findings of this study?**

- In 132 IBD/non‐IBD gastrointestinal diseases matched pairs, 27/62 (43.6%) faecal VOCs were statistically significantly decreased in Crohn's disease (CD), ulcerative colitis (UC) or both.
- Propan‐1‐ol was increased in CD and extensive colitis and decreased with treatment. Phenol was increased in CD and fell with treatment in CD and UC. Oct‐1‐en‐3‐ol was increased in CD and UC and fell with treatment in UC.
- Measurement of faecal VOCs provides insights into disease pathogenesis and has potential for the non‐invasive diagnosis and monitoring of IBD.

## INTRODUCTION

Volatile organic compounds (VOCs) are carbon‐based, low molecular mass molecules that contribute to the odour of biological samples. In the gut, VOCs result mainly from the metabolism of the gut microbiota and the intestinal mucosa and their abundance changes in intestinal disease.^1^ In inflammatory bowel disease (IBD), a loss of gut microbial diversity is correlated with disease severity and with greater derangement in Crohn's disease (CD) than ulcerative colitis (UC).^2^

Faecal VOCs are relatively stable over time within and between individuals despite day‐to‐day variations in diet and are not affected by optimized freezing/storage of stool samples.^3^ In adults, most of whom were taking medication at the time of sampling, faecal VOCs differ between diarrhoea‐predominant irritable bowel syndrome and active CD and UC, between Crohn's colitis and UC^4^ and between active and inactive CD and UC.^5^ In small studies in children, faecal^6^ and urinary^7^ VOCs were altered in active IBD.

We characterized faecal VOCs in children newly presenting with suspected IBD. The objectives were to compare IBD with controls and assess how faecal VOCs were related to disease sub‐type, disease severity, the region of bowel affected and response to treatment in IBD. We also consider how characterisation of faecal VOCs may provide insights into pathogenesis in IBD.

## MATERIALS AND METHODS

### Study design and settings

We undertook a prospective, case‐control study of children attending paediatric gastroenterology clinics in Alder Hey Children's Hospital, Liverpool; Bristol Royal Hospital for Children, Bristol; and Birmingham Children's Hospital, Birmingham, UK.

### Screening, recruitment, sample collection and clinical management

Children with suspected IBD were identified from referral letters and asked to bring a stool sample when attending the clinic (Table S1 and Supplementary Methods). If suspected IBD was confirmed following clinical assessment, a research nurse provided written and verbal information about the study. Children were excluded if they had an established diagnosis of IBD or another significant intestinal disorder or had already started treatment for IBD including exclusive enteral nutrition. Signed, informed consent was secured from young people aged 16 years, or the parent or guardian in younger children.

Clinical diagnosis was assessed at 3 months follow‐up by review of medical records and a further stool sample requested in those with IBD. IBD was diagnosed,^8^ managed,^9^, ^10^ and disease distribution^11^ and activity^9^ assessed following established clinical guidelines. Similarly, appropriate investigations were performed to diagnose non‐IBD disorders (e.g., endoscopy and small intestinal biopsy in coeliac disease (Supplementary Methods)). Children in whom a diagnosis other than IBD was made served as controls.

To maximise relevance to clinical practice, each IBD case was matched by age (±6 months), sex and recruitment site to a non‐IBD control. Children in whom the diagnosis of IBD could not be either confirmed or refuted at follow‐up were excluded from the analysis.

### Data extraction

Demographic, clinical and laboratory information was extracted from medical records and collected by questionnaire. For diet, we asked for major dietary modifications (e.g., vegetarian diet).

### Sample storage and analysis

Stool aliquots were stored at −80°C and shipped frozen to the University of Liverpool. Faecal VOCs were characterized by gas chromatography–mass spectrometry (GC‐MS). Faecal calprotectin (FC) was measured at each hospital according to their usual practice (Supplementary Methods). Laboratory staff were blinded to the patient's diagnosis and response to treatment in those with IBD.

### Sample size calculation

This study analysed all case/control pairs from a study evaluating an electronic nose in the differentiation of IBD from other gastrointestinal disorders (to be reported separately), which aimed to recruit a total of 286 children with suspected IBD.

### Data processing

Raw data were processed by Automated Mass Spectral Deconvolution System (AMDIS‐version 2.71, 2012) coupled to the National Institute of Standards and Technology mass spectral library (version 2.0, 2011) to putatively identify VOCs (Supplementary Methods).

### Statistical analysis

Continuous demographic, clinical and environmental variables were summarized in subjects as mean and standard deviation if normally distributed and median and inter‐quartile range (IQR) if non‐normally distributed (SPSS Statistics; version 26, IBM). Categorical variables were summarized according to the absolute frequency and percentage of subjects. The denominator is the number of subjects with data available unless stated otherwise.

The number of VOCs detected in different groups prior to sparse feature removal (Supplementary Methods) and imputation of missing values were compared using the Wilcoxon signed‐rank test or, where data were normally distributed (confirmed by Shapiro–Wilk test), a paired *t*‐test and variability in numbers by the coefficient of variation (CV; R, version 3.6). *T*‐tests and one‐way analysis of variance were used to compare VOC abundance according to disease severity, distribution and response to treatment. To maximise the number of observations, we combined UC and IBD‐unspecified (IBD‐U) for the analysis of disease severity and distribution. A paired *t*‐test was used to compare IBD baseline and follow‐up samples (online software tool Metaboanalyst version 5.0, https://www.metaboanalyst.ca). Logistic least absolute shrinkage selection operator (LASSO) was performed using the R package to assess the association between VOCs abundance and FC at baseline and dietary therapy in CD at follow‐up.

Given the similar clinical presentation and response to treatment in children with IBD in both sexes,^12^ we have not reported findings disaggregated by sex. In this exploratory study, *p* < 0.05 was accepted to show statistically significant differences with no correction for multiple comparisons.

## RESULTS

**FIGURE 1:**
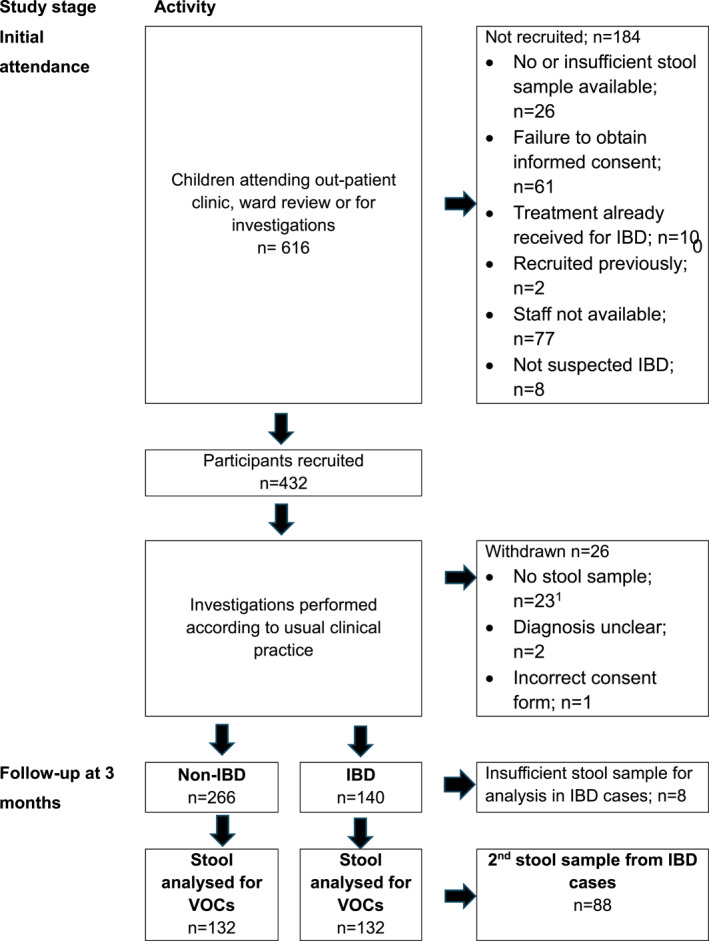
Flow diagram. ^1^Children who had intended to provide a stool sample were withdrawn if an inadequate or no sample was provided.

Between June 2017 and June 2020, 432/616, children attending gastroenterology clinics with suspected IBD were recruited (Figure 1). Twenty‐six children were withdrawn, mainly because of inability to obtain a stool sample. In the remaining children, 132/140 children diagnosed with IBD and with sufficient stool for VOC analysis were matched with a non‐IBD control.

Demographic, clinical, and environmental variables were broadly similar amongst IBD and non‐IBD children and IBD sub‐types although there was a greater proportion of South Asian children amongst the IBD cases and those recruited in Birmingham (Table 1 and Tables S2 and S3). Amongst the IBD cases, 78/132 (59%) had CD, 38 (29%) had UC and 16 (12%) had IBD‐U (Table S4). Common diagnoses amongst the non‐IBD controls were functional gastrointestinal disorders (68, 51.5% children: 25 functional abdominal pain, 24 irritable bowel syndrome, 19 functional constipation) and coeliac disease (7; 5.3%).

**TABLE 1:** Demographic variables and diet according to diagnosis.

Variable	IBD cases				Controls
	CD	UC	IBD‐U	Total	*N* = 132
	*N* = 78	*N* = 38	*N* = 16	*N* = 132	
Age (y)					
Median	13.6	12.9	13.3	13.3	12.9
(IQR)	(10.2–14.9)	(10.2–14.4)	(10.0–15.3)	(10.2–14.7)	(10.1–14.7)
Range	4.6–16.2	5.1–16.3	7.1–16.3	4.6–16.3	3.7–16.8
Female *n* (%)	33 (42.3)	13 (34.2)	5 (31.3)	51 (38.6)	51 (38.6)
Ethnicity *n* (%)^a^					
• White	59 (75.6)	25 (65.8)	15 (93.8)	99 (75.0)	115 (87.1)
• Asian	13 (16.7)	10 (26.3)	1 (6.3)	24 (18.2)	11 (8.3)
• Other	6 (7.7)	3 (7.9)	0 (0.0)	9 (6.8)	6 (4.5)
Diet *n* (%)^b^					
• Standard	71 (91.0)	32 (84.2)	15 (93.8)	118 (89.4)	116 (87.9)
• Vegetarian	1 (1.3)	3 (7.9)	1 (6.3)	5 (3.9)	5 (3.8)
• Gluten free	3 (3.9)	1 (2.6)	0 (0)	4 (3.2)	5 (3.8)
• Dairy/lactose free	2 (2.6)	1 (2.6)	0 (0)	3 (2.4)	2 (1.5)

Abbreviations: CD, Crohn’s disease; IBD‐U, IBD‐unspecified; UC, ulcerative colitis.

^a^ The proportion of South Asians with IBD was significantly greater than Europeans (*p* < 0.036).

^b^ The number in each dietary category was similar in each group (*P* = not significant).

Across all samples, 62/152 identified faecal VOCs remained after filtering for sparse features. Ketones (*n* = 13), fatty acids (11), and aldehydes (11) were the most common VOCs identified (Table S5). There was no clustering for hospital in a principal component analysis of VOC abundances (Figure S1). VOC abundances were not significantly different between hospitals (Kruskal–Wallis, *p* > 0.05). The numbers of VOCs detected were significantly lower in samples recruited from Alder Hey Children's Hospital (median 37, IQR 11) compared to those recruited from Bristol Children's Hospital only (median 40, IQR 10, Kruskal–Wallis, *p* = 0.034) (Table S6). Collectively, these results suggest minimal differences in VOCs between hospital sites.

### Faecal VOCs in Crohn's disease

The number of VOCs detected in children with CD (median 39, IQR 13) and matched controls (median 40, IQR 11) was similar (Wilcoxon signed‐rank test *p* = 0.282). However, the CV was higher for CD (28%) than controls (20%; Figure 2a). 5/62 (8.1%) VOCs had higher and 9 (14.5%) had lower mean abundance in CD compared to controls (*p* < 0.05; Figure 2b; Table S7).

FIGURE 2Faecal VOCs in CD. (a) Number of VOCs at baseline in matched controls and CD according to disease activity (defined by wPCDAI) and distribution (defined by the Paris classification: L1—distal 1/3 ileum with or without limited cecal disease; L2—colonic disease; L3—ileocolonic disease; C = Control). (b) Abundance of VOCs at baseline that differed significantly between CD and matched controls. (c) VOCs which altered significantly with disease activity (as in panel (a)) in CD at baseline (one‐way ANOVA, *p* < 0.05); results of statistical comparisons are listed in Table S8. (d) VOCs which altered significantly with disease distribution (as in panel (a)) at baseline (one‐way ANOVA, *p* < 0.05); results of statistical comparisons are listed in Table S10. (e) Control (*n* = 53), baseline (BL, *n* = 53), active at follow‐up (AFUP, *n* = 29), remission at follow‐up (RFUP, *n* = 24). Propan‐1‐ol, phenol and ethanol were significantly raised in CD compared to control at baseline and reduced post‐treatment (*p* < 0.05, paired *t*‐test). ANOVA, analysis of variance; CD, Crohn's disease; VOCs, volatile organic compounds.

**Figure:**
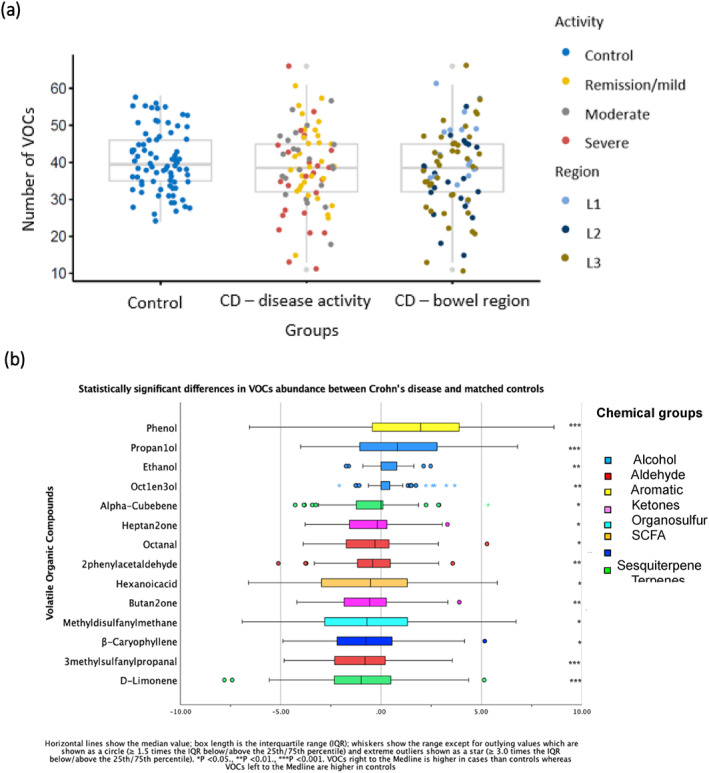

**Figure:**
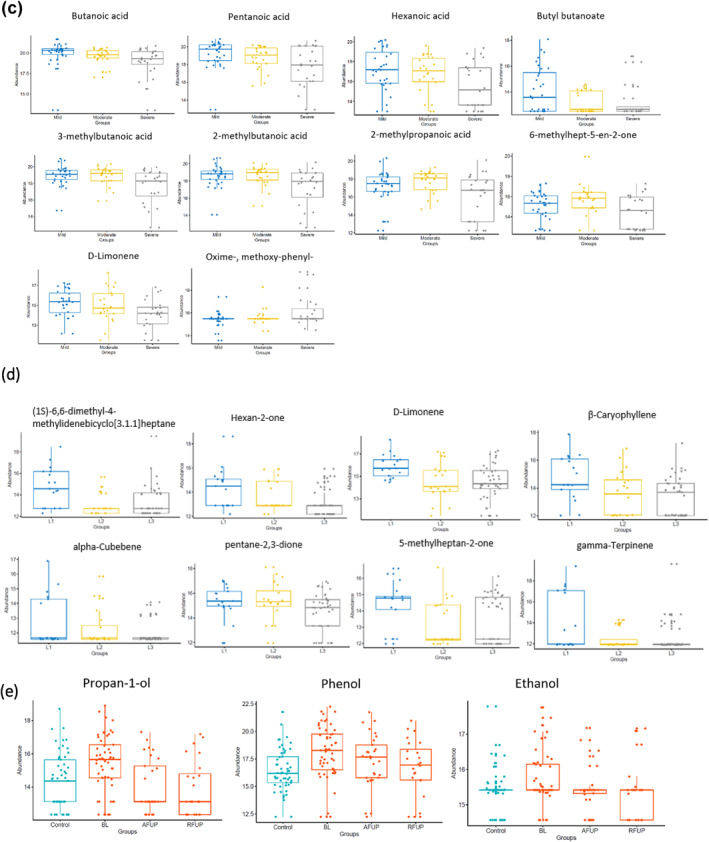

The number of VOCs detected did not differ according to disease severity (Figure 2a; Table S4, Kruskal–Wallis test *p* = 0.23). However, the abundance of 10 VOCs varied significantly with disease severity, with most decreasing as severity increased (Figure 2c; Table S8), including a short chain fatty acid (SCFA; butanoic acid, *p* = 0.030) 2 medium chain fatty acids (MCFA; pentanoic acid, hexanoic acid, *p* = 0.008, *p* = 0.020, respectively), and 3 branched chain fatty acids (BCFA; 2‐methylpropanoic acid, 3‐methylbutanoic acid, 2‐Methylbutanoic acid, *p* = 0.017, 0.002, 0.002, respectively).

Median (IQR) FC at baseline in CD was 1216 μg/gm stool (IQR 600–1800); only phenol was significantly positively associated with FC (Table S9).

Although the number of VOCs did not differ significantly according to disease distribution (Figure 2a; Table S4, Kruskal–Wallis test *p* = 0.090), the abundance of 8 VOCs varied according to distribution with 6 VOCs associated with ileal (LI) disease (Figure 2d, Table S10; *p* < 0.05).

Fifty‐three children provided a stool sample after 3 or more months (Table S4). The mean number of VOCs at follow‐up (38, SD 11; paired *t*‐test *p* = 0.42) and the variability in VOC numbers (CV = 25%) were similar to baseline (mean 37, SD 9, CV = 25%). The abundance of 4‐ethylphenol was significantly increased (paired *t*‐test *p* = 0.02), but four VOCs significantly reduced at follow‐up (Table S11; *p* < 0.05). Propan‐1‐ol, phenol and ethanol significantly increased at baseline compared to controls (paired *t*‐test *p* ≤ 0.001, <0.001, 0.002, respectively, Table S7), subsequently fell to levels similar to, or below, that of controls post‐treatment (Figure 2e). Propan‐1‐ol reduced regardless of disease activity at follow‐up and phenol showed a reducing trend in remission (*p* = 0.12; Figure 2e; Table S11).

At follow‐up, 19 (35.8%) children with CD were receiving nutritional therapy; some ketones, medium chain fatty acids, and an alcohol had a weak negative correlation with dietary therapy (Table S12).

### Faecal VOCs in ulcerative colitis and colitis (UC and IBD‐U combined)

**FIGURE 3:**
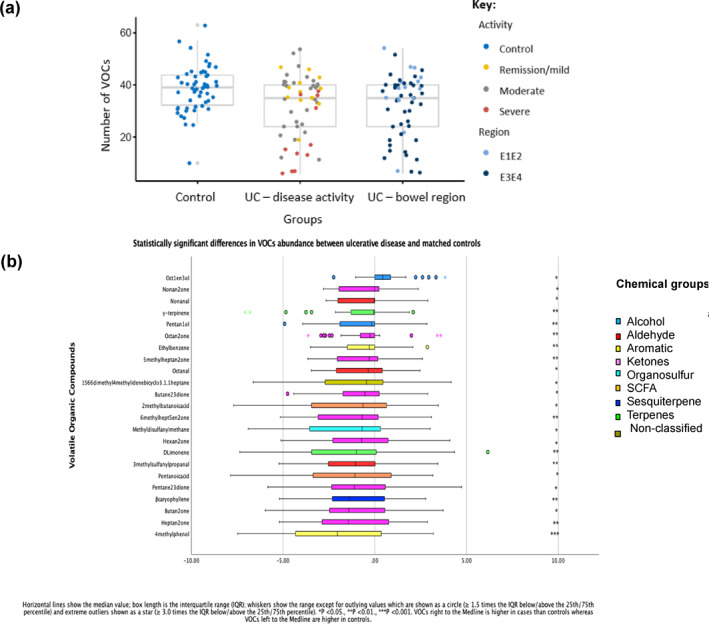
Faecal VOCs in UC. (a) Number of VOCs at baseline in matched controls and UC according to disease activity defined by PUCAI and distribution defined by the Paris classification: E1E2 = left‐sided colonic inflammation, E3E4 = more extensive colonic inflammation; C = Control. (b) Abundance of VOCs at baseline that differed significantly between UC and matched controls. PUCAI, paediatric ulcerative colitis activity index; UC, ulcerative colitis; VOCs, volatile organic compounds.

Significantly fewer VOCs were detected in UC (median, 35 IQR 18) than in matched controls (median 38, IQR 9; Wilcoxon rank‐sum test, *p* = 0.026) and VOC numbers were more variable in UC (CV = 40%) than controls (CV = 18%; Figure 3a) or CD (CV = 28%). The mean abundance of 22/62 (35.5%) VOCs was lower, whereas oct‐1‐en‐3‐ol was higher in UC than in controls (Wilcoxon Signed Rank Test *p* = 0.012; Figure 3b; Table S13).

**FIGURE 4:**
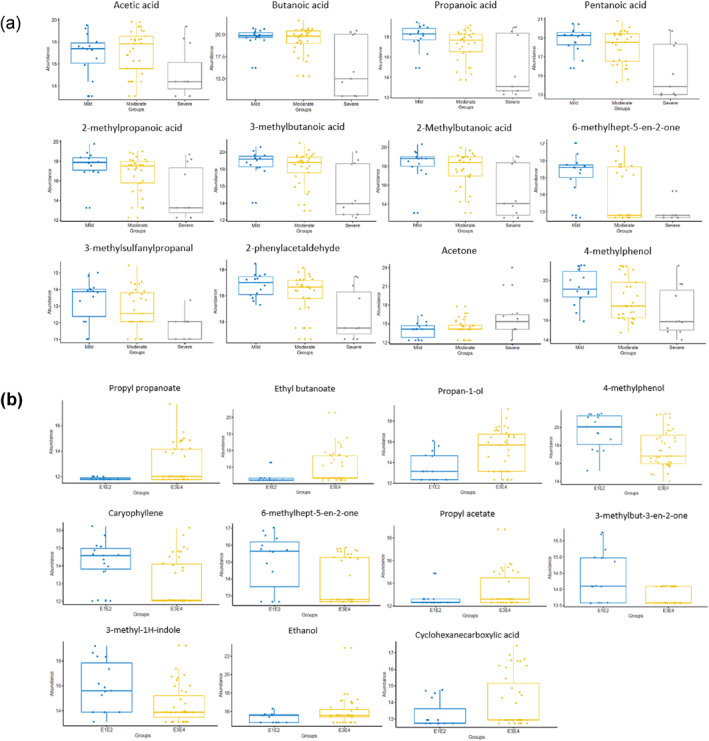
Faecal VOCs in colitis at baseline (UC and IBD unclassified combined). (a) VOCs which altered significantly with disease activity (as in Figure 3a) in colitis at baseline (one‐way ANOVA, *p* < 0.05); results of statistical comparisons are listed in Table S14. (b) VOCs which altered significantly with disease distribution (as in Figure 3a) at baseline (one‐way ANOVA, *p* < 0.05); results of statistical comparisons are listed in Table S15. IBD, inflammatory bowel disease; ANOVA, analysis of variance; UC, ulcerative colitis; VOCs, volatile organic compounds.

There were fewer VOCs identified in severe (median 15, IQR 24) compared to moderate (median 37, IQR 16, pairwise Wilcoxon rank sum test, *p* = 0.008) and mild disease/remission (median 39, IQR 8, *p* = 0.004) colitis (Table S4). The abundance of 18 (29.0%) VOCs decreased as disease worsened (*p* < 0.05) whereas acetone was increased in more severe disease (one‐way analysis of variance *p* = 0.014; Figure 4a; Table S14).

Median (IQR) FC at baseline in colitis was 1748 μg/gm stool (IQR 600–1800). Ethanol was positively, and 6‐methylhept‐5‐en‐2‐one negatively associated with FC (LASSO = 20.69, −99.80, respectively; Table S9). There were no associations between FC and VOC abundances when controls only were analysed in LASSO regression.

Numbers of VOCs showed a trend of being higher in distal (E1 + E2, median, 39, IQR 7) than in more extensive (E3 + E4, median 33, IQR, 5) inflammation (Figure 3a, Wilcoxon rank sum test, *p* = 0.052). The abundance of 11 (17.7%) VOCs varied significantly with disease extent, with roughly half associated with distal disease and half with extensive disease (Figure 4b; Table S15; *p* < 0.05).

In the follow‐up of 27 children with UC, the number of VOCs (mean 34, SD 12, paired *t*‐test, *p* = 0.96) and the variability of VOC numbers (CV = 35%) was similar to baseline (mean 34, SD 12).

**FIGURE 5:**
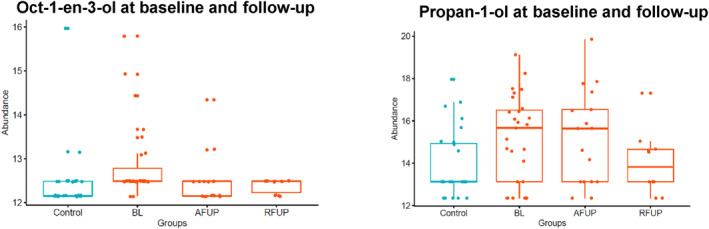
Oct‐1‐en‐3‐ol and propan‐1‐ol in UC and controls at baseline and UC at follow‐up. Oct‐1‐en‐3‐ol and propan‐1‐ol were raised in UC compared to controls at baseline and significantly reduced post‐treatment (*p* < 0.05; paired *t*‐test). Control (*n* = 27), baseline (BL, *n* = 27), active at follow‐up (AFUP, *n* = 17), remission at follow‐up (RFUP, *n* = 10). UC, ulcerative colitis.

Oct‐1‐en‐3‐ol was significantly increased in UC compared to controls at baseline (*p* = 0.012; Table S13) and decreased to levels similar to controls for active disease and remission at follow‐up (Figure 5). Similarly, propan‐1‐ol tended to be higher in cases than controls at baseline (*p* = 0.054; Table S13) and at follow‐up decreased significantly in remission (10 pairs, paired *t*‐test, *p* = 0.046) but not in active disease (17 pairs, paired *t*‐test, *p* = 0.41; Figure 5). The abundance of phenol fell significantly from baseline and was lower in abundance at follow‐up even in children with active disease (Paired *t*‐test *p* = 0.02; Table S16). No children with UC were receiving therapeutic feeds at follow‐up.

### Faecal VOCs in Crohn's disease versus ulcerative colitis at baseline

**FIGURE 6:**
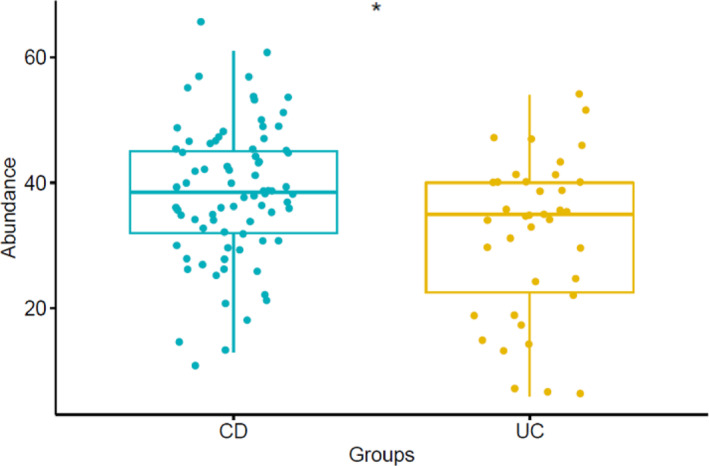
VOC profile comparisons between CD and UC. Number of VOCs in CD and UC. CD, Crohn’s disease; UC, ulcerative colitis; VOCs, volatile organic compounds.

The number of VOCs was higher in CD (median 39, IQR 13) than in UC (median 35, IQR 18; Wilcoxon rank sum test *p* = 0.013; Figure 6). The abundance of 15 VOCs, mainly ketones and fatty acids, was higher in CD, whereas 3‐methylbutanal was increased in UC (Student's *t*‐test, *p* < 0.05, Table S17).

### Faecal VOCs in small versus all large bowel disease

**FIGURE 7:**
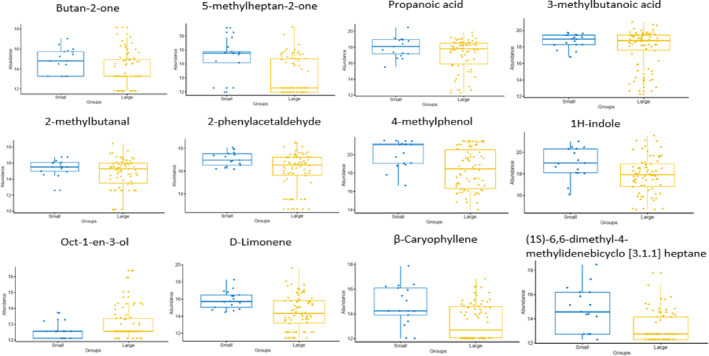
Faecal VOCs comparisons of small and large bowel disease. In total, 25 VOCs were significantly different (Student's *t*‐test); a subset of 12 are shown to represent VOCs which altered across the chemical classes. VOCs, volatile organic compounds.

Twenty‐four VOCs were increased in the small bowel and oct‐1‐en‐3‐ol was increased in the large bowel disease (Student's *t*‐test *p* = 0.009; Table S18). Figure 7 represents VOCs which altered across the chemical classes.

A summary of the VOCs that differed significantly in abundance according to clinical parameters in IBD is shown in Table S19.

## DISCUSSION

This is the largest GC‐MS study of faecal VOCs in an inception cohort of children with IBD and matched controls with non‐IBD gastrointestinal disorders. There were clear differences between IBD and controls in the number of VOCs in UC, and in the abundance of many VOCs in both UC and CD. Some of these differences were associated with disease activity and, to a lesser extent, disease distribution, and also with response to treatment in IBD cases. The sources of VOCs highlighted in our study and evidence from other studies of IBD are summarised in Table S20.

### Differences between IBD and matched controls at baseline

The mean number of faecal VOCs was significantly lower in UC than in controls. The abundance of 12 VOCs was significantly lower in UC, three in CD and six in both CD and UC. Given that the metabolism of the gut microbiota is the main source of faecal VOCs,^2^ these findings are consistent with the reduced microbial diversity and dysbiosis reported in IBD in adults^2^ and children.^13^

In contrast to the overall pattern of reduced VOCs abundance in IBD, four VOCs were in greater abundance in CD than in controls (three alcohols and phenol). One of these alcohols, oct‐1‐en‐3‐ol, was also in greater abundance in UC suggesting an increase in some bacterial taxa in IBD^14^ or the presence of fungi which frequently produce 8‐carbon compounds.^15^

### Disease severity

At baseline in UC, both the number and abundance of VOCs were reduced in more severe disease. In both CD and UC, several fatty acids were less abundant in more severe disease. Fatty acids were similar in IBD and controls at baseline, indicating that the change in these compounds may be related to disease severity; a decrease in SCFA‐producing bacteria with increased severity in new‐onset paediatric UC has been reported previously.^16^ In CD, differences in short and medium chain fatty acids have been observed between active and inactive cases^17^ but the stepwise reduction with increasing disease severity that we have reported for several VOCs is a new finding. We observed lower abundance of BCFA in UC and CD and also 4‐methylphenol and phenol in UC, all products of amino acid fermentation. BCFAs are produced mainly by *Bacteroides* and *Clostridium* fermentation of branched‐chain amino acids (valine, leucine and isoleucine^18^). De Preter et al. also noted a reduction in the aromatic compounds 4‐methylphenol and methyl‐indole in active UC.^17^ These findings are consistent with a change in the metabolism or composition of the intestinal microbial community in active IBD.

Although not associated with disease activity in CD, the positive association between phenol and FC at baseline is consistent with the reduction in phenol abundance with clinical improvement at follow‐up. 6‐methylhept‐5‐en‐2‐one was lower in more severe colitis and also negatively associated with FC. These two VOCs may have a role in monitoring disease activity.

### Disease distribution in IBD

Studies evaluating faecal VOCs according to disease distribution are scarce. The abundance of 25 VOCs differed significantly between small and large bowel disease, with 24 VOCs (predominantly ketones/diketones, fatty acids, some aldehydes and aromatic compounds) increasing in small bowel disease. In contrast, the alcohol oct‐1‐en‐3‐ol was increased in large bowel disease. Previous metabolomic studies were able to distinguish between predominantly ileal and colonic CD and indicated that metabolites of fatty acids, bile acid, tyrosine and phenylalanine biosynthesis may be of importance in the pathogenesis of CD.^19^ Recently, Notararigo et al. reported higher levels of homoserine‐methionine and isobutyrate (2‐methylpropanoic acid) in serum in ileocolonic CD.^20^ The abundance of specific VOCs may have clinical utility in identifying disease distribution in IBD.

### Differentiating IBD sub‐type

Strikingly, 17 VOCs differed significantly in abundance between CD and UC, with most increased in CD. These were mainly ketones/diketones, fatty acids and aromatic compounds. The diketone 6‐methylhept‐5‐en‐2‐one and the aromatic compound 4‐methylphenol were lower in abundance in UC than controls at baseline and in severe and extensive colitis and may be particularly helpful in differentiating disease subtypes. Furthermore, 3‐methylbutanal was increased in UC compared with CD and was not significantly associated with any other comparisons; therefore, it may also be a useful marker to distinguish between subtypes.

### Response to treatment in IBD

The reduced number and abundance of many VOCs at baseline persisted at follow‐up, suggesting persistent dysbiosis despite clinical improvement in many children. In paediatric IBD in the Netherlands, treatment did not result in microbiota recovery to levels in healthy controls.^21^ The weak associations between VOCs and dietary therapy in CD suggest that the changes in VOCs following treatment reflect differences in disease status rather than a change in gut flora resulting from nutritional therapy. Changes in specific VOCs to levels found in controls may have utility as treatment targets in IBD.

### Specific VOCs of interest

The difference in abundance compared to controls at baseline, association with disease severity, distribution and sub‐type and change in abundance following treatment support the role of some specific VOCs in pathogenesis and monitoring response to treatment.

The abundance of the alcohol propan‐1‐ol was significantly increased in CD and possibly also in UC (*p* = 0.054) and with more extensive disease in UC. Levels reduced to those similar to controls after treatment in CD and showed a similar trend in UC in remission. Resulting from the degradation of the amino acid threonine by *Escherichia coli*, other *Enterobacteriaceae* ^22^ and *Clostridium* sp,^23^ propan‐1‐ol is considered to be damaging to the gut.^24^ Found to be related to active IBD previously,^25^ propan‐1‐ol is a promising compound in breath, faeces and urine for both the diagnosis and monitoring of IBD.^4^, ^25^, ^26^

Another alcohol, oct‐1‐en‐3‐ol, was notable as the only VOC in greater abundance in UC and was also raised in CD and in large bowel disease at baseline. Post‐treatment, oct‐1‐en‐3‐ol reduced significantly in UC but not in CD. In a study in adults, Ahmed et al. also reported raised oct‐1‐en‐3‐ol in CD.^5^ We also observed that ethanol was in significantly greater abundance in CD and severe colitis at baseline and fell following treatment in CD. Oct‐1‐en‐3‐ol, a by‐product of the enzymatic breakdown of linoleic acid,^27^ and ethanol are associated with fungal overgrowth.^27^, ^28^, ^29^ Although Ott et al. reported a greater diversity in fungi in colonic biopsies in CD compared with controls,^30^ CD in clinical remission was associated with greater fungal diversity in faeces where fungal profiles clustered with *Candida* spp.^31^ Our findings support further research regarding the role of intestinal fungi in IBD.

Phenol was in greater abundance in CD and fell with treatment to levels seen in controls in both CD and UC, with levels tending to be lower in IBD cases that achieved remission. Increased phenol abundance in stools has been reported in adults with CD, UC and also irritable bowel syndrome.^25^ Phenol arises from the microbial degradation of tyrosine and tryptophan^24^ and is produced by many intestinal bacterial species including *Enterobacteriaceae* and *Clostridium clusters I*, *XI*, and *XIVa*.^32^ Phenol is considered to be damaging to the gut^24^ exhibiting cytotoxicity and increased paracellular permeability in vitro and impaired integrity of the intestinal epithelium and the viability of intestinal epithelial cells.^33^, ^34^

### Strengths and limitations

Recruiting children with non‐IBD gastrointestinal disorders as the comparison group, rather than healthy controls, increase the relevance of our findings to clinical practice. The matching of the case and controls by age, sex and recruitment site and the similarity in demographic variables, including diet, and exposure to antibiotics amongst the cases and controls, suggest that the differences in faecal VOCs were due to disease status rather than environmental factors influencing the gut microbiota. The recruitment of children at initial presentation excluded the effect of IBD drug treatment on VOCs, which may occur in studies of established disease.

We were not able to test our findings in a validation cohort. Some children did not provide follow‐up stool samples, limiting our ability to evaluate the effects of response to treatment. Although we have identified faecal VOCs that may cause or be a consequence of intestinal damage, further work is needed to quantify these compounds to assess their importance in IBD pathogenesis. Using a single assay for FC with a wide range of measurements may have improved our assessments of the association between VOCs and disease severity. Aligning metabolomic studies and therapeutic trials would help explain how VOCs are regulated and hold the potential for developing a biomarker to predict clinical response to specific IBD therapies as we have proposed for irritable bowel syndrome.^35^ Future work should also investigate the source of the VOCs of interest using genomics tools.

A further replication cohort would add strength to the current work. Future studies might target VOCs that changed most with disease activity and might serve as biomarkers, and the role of fungi in UC, given our findings regarding octen‐3‐ol. Finally, longer follow‐up to assess how VOCs change between remission and later relapse will strengthen the associations that we have reported.

## CONCLUSIONS

Characterisation of faecal VOCs in IBD provides insights into the pathogenesis that may inform the development of novel interventions. Despite the challenges often faced in the reproducibility of metabolomics studies, we have reproduced the findings of other studies that propan‐1‐ol, phenol and oct‐1‐en‐3‐ol are markers of active IBD. Further work is needed to confirm if the number of unique faecal VOCs and change in abundance are functional representatives of the gut microbiota and explore their clinical utility in differentiating IBD sub‐types, determining disease severity and distribution and monitoring response to treatment.

## AUTHOR CONTRIBUTIONS

The following authors made substantial contributions to the conception and design of the study (SA, CP, DW), acquisition of clinical data (SA, RM, CS), laboratory analyses (RS, SB, CP) and interpretation of data (all authors). Drafting the article (SB); revising the article critically for important intellectual content (all authors). Final approval of the version to be submitted (all authors). None of the authors had any writing assistance.

## CONFLICT OF INTEREST STATEMENT

The authors have no conflicts of interest to declare.

## ETHICS APPROVAL

The study was approved by the North West—Preston Research Ethics Committee, UK (reference 17/NW/0333) and the NHS Health Research Authority (Integrated Research Application System project ID 223199).

## STUDY REGISTRATION

The study is registered with the International Standard Randomised Controlled Trials Number Register: 11314352.

## Supporting information

Supporting Information S1

## Data Availability

The study data, analytical methods, and study materials will be made available to other researchers on reasonable request to the corresponding author.
