# Supplementary material for: Faecal volatile organic compounds differ according to inflammatory bowel disease sub‐type, severity, and response to treatment in paediatric patients
Source: United European Gastroenterol J. 2024 Jun 22;12(6):780–92. doi: 10.1002/ueg2.12603 (PMC11249809; doi:10.1002/ueg2.12603)
Supplement: Supplementary file 1 — Supporting Information S1 [file UEG2-12-780-s001.docx]

**Faecal Volatile Organic Compounds in an Inception Cohort of Paediatric Inflammatory Bowel Disease. Supplementary materials.** Dr Salma Belnour et al.

**Supplementary methods**

**Diagnosis and classification of IBD**

The distribution of intestinal inflammation in IBD and associated manifestations (e.g. presence of fistulas in CD) were recorded according to the Paris classification.^[[1]](#footnote-1)^ The severity of CD was graded according to the weighted Pediatric Crohn’s Disease Activity Index (wPCDAI) with ≤12.5 in the absence of corticosteroid treatment, 12.5‐40, 42.5‐57.5 and >57.5 indicated clinical remission, mild, moderate, and severe disease respectively^2^. For the Pediatric Ulcerative Colitis Activity Index (PUCAI), <10 in the absence of corticosteroid treatment, 10-34, 35-64, 65 or above indicated clinical remission, mild, moderate, and severe disease respectively.^[[2]](#footnote-2)^

**Stool sample collection**

Following review of the referral letter (STable 1), children or parents/guardians were asked to collect 2-3 scoops of stool using a FecesCatcher (Alpha Laboratories, Eastleigh UK) and spatula, or an equivalent volume of liquid stool, into a hard plastic Sterilin tube either the day before or the morning of the clinic visit as part of routine care. The tube was double-bagged using zip-lock plastic bags. If collected the day before clinic, the sample was stored in the home freezer and then brought to the clinic at ambient temperature. A stool sample was collected during review from children referred directly to the ward. Short term storage at room temperature, from example while travelling to the hospital, does not alter headspace VOCs significantly.^[[3]](#footnote-3)^

**Characterisation of faecal VOCs by GC-MS**

Faecal VOCs were characterized using a Perkin Elmer Clarus 500 gas chromatography/ mass spectrometry system (Beaconsfield, UK). Headspace VOCs were extracted from 100mg faecal samples, in 10 ml headspace vials, using a divinylbenzene-carboxen-polydimethysiloxane solid phase micro-extraction automated by a CombiPAL system (CTC Analytics, Switzer- land). The gas chromatography–mass spectrometry conditions were as described previously.^[[4]](#footnote-4)^

**Measurement of faecal calprotectin (FC)**

In Liverpool, FC was measured initially by ELISA *Bühlmann*fCAL Calprotectin kit for DS2 and then subsequently by an EliA Calprotectin fluorescence enzyme immunoassay (maximum value for both assays = 2100μg/g stool); in Birmingham by Bühlmann Calprotectin ELISA kit (two kits used with maximum values = 600μg/g and 1800μg/g stool) and in Bristol by ELISA Bühlmann fCAL Calprotectin kit for DS2. Stool samples that were rejected by hospital laboratories (e.g., because of the presence of blood), were transported to Liverpool and FC was measured by Faecal Calprotectin Elisa Bühlmann fCAL EK-CAL2 (maximum value = 1800 μg/g stool). Samples with FC values above the range measured were allocated the maximum value according to the assay used.

**Data processing**

For the GC-MS analysis, the R package Metab^[[5]](#footnote-5)^ was used to align data in R (version 3.6). Sparse VOCs that were present in less than 20% of either IBD or control samples were removed and not included in analyses. Such sparse data reflects infrequent VOCs that usually arise from diet. These compounds do not aid modelling and increase the risk of missing a signal when adjusted for the number of variables. Compounds below the level of detection by GC (“missing values”), were imputed before analysis. We favour “half-minimum values” for such imputation. Data were then normalised by log-transformation (natural log). Batch correction was undertaken using the R function ComBat, package sva^6^ to account for sample processing before and after a GC-MS filament change.

**SFigure 1: PCA of VOCs according to recruitment site**


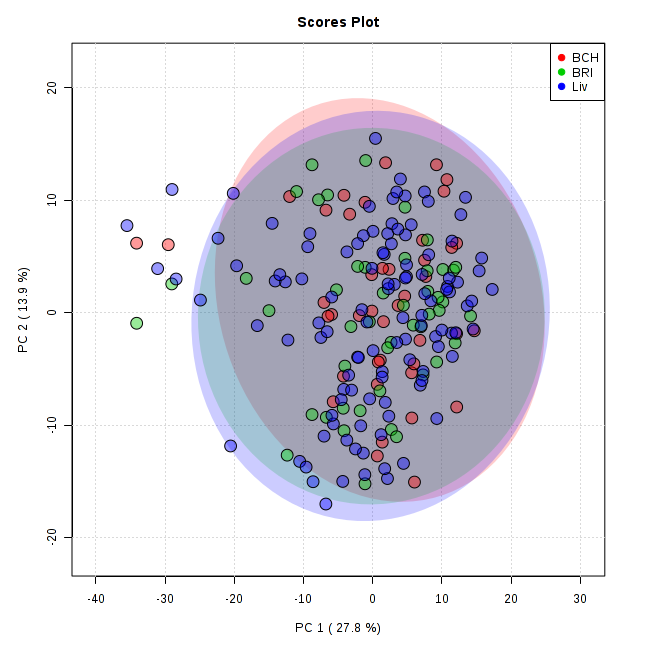


**STable 1:** **Schedule of Procedures**

| **Procedures** | **Visit** | |
| --- | --- | --- |
|  | **Screening/**  **Baseline**  **Visit 1** | **Follow Up/**  **3 months**  **Visit 2** |
| Informed consent | x |  |
| Demographics | x |  |
| Medical history | x |  |
| Physical examination | x |  |
| Eligibility assessment | x |  |
| Arrangements for clinical investigations as required for routine clinical management (e.g., faecal calprotectin, endoscopy) | x |  |
| Faecal sample collected for VOCs analysis | x |  |
| Concomitant medications | x |  |
| Confirmation of diagnosis (all children) |  | x |
| In confirmed IBD: disease classification, distribution, severity at recruitment (from medical records) |  | x |
| In confirmed IBD: previous and current treatment; concomitant medications |  | x |
| In confirmed IBD: assessment of clinical remission (disease activity scores) |  | x |
| In confirmed IBD: faecal sample collected and analysed for VOCs and calprotectin |  | x |

**STable 2: Demographic, clinical and environmental variables at recruitment according to diagnosis^1^**

| **Variable** | **IBD cases** | | | | | **Controls**  N=132 |
| --- | --- | --- | --- | --- | --- | --- |
|  | **CD**  N=78 | **UC**  N=38 | | **IBD-U**  N=16 | **TOTAL**  N=132 |  |
| **No. siblings at home** |  |  | |  |  |  |
| Median (IQR)  range | 2 (2-3)  0-6 | 2 (1-3)  1-6 | 2 (1-2)  0-4 | | 2 (1-3)  0-6 | 2 (1-3)  0-6 |
| - none | 1 (1.3) | 0 (0) | 2 (12.5) | | 3 (2.3) | 1 (0.8) |
| - one | 14 (17.9) | 16 (42.1) | 3 (18.8) | | 33 (25.0) | 36 (27.3) |
| - two or more | 63 (80.8) | 22 (57.9) | 11 (68.9) | | 96 (72.7) | 95 (72.0) |
| **Pets in the home** |  |  |  | |  |  |
| Median (IQR)  range | 1. (0-1)   0-5 | 1.0 (0-2)  0-15 | 1.0 (0-1)  0-3 | | 1.0 (0-2)  0-15 | 1.0 (0-2)  0-19 |
| - No pets | 42 (53.8) | 15 (39.5) | 7 (43.8) | | 64 (48.5) | 47 (35.6) |
| - 1 or more pets | 36 (46.2) | 23 (60.5) | 9 (56.3) | | 68 (51.6) | 85 (63.6) |
| - one or more dogs | 23 (29.7) | 15 (39.2) | 6 (37.6) | | 44 (32.5) | 62 (48.3) |
| - one or more cats | 12 (15.5) | 12 (31.4) | 2 (12.6) | | 26 (20.4) | 31 (72.7) |
| **Smoking in household**^2^ | 9 (11.6) | 8 (21.1) | 1 (6.3) | | 18 (13.6) | 25 (18.9) |
| **Residence type** |  |  |  | |  |  |
| - urban | 62 (79.5) | 33 (86.8) | 11 (68.8) | | 106 (80.3) | 111 (84.1) |
| - rural | 16 (20.5) | 5 (13.2) | 5 (31.3) | | 26 (19.7) | 21 (15.9) |
| **Antibiotic use** | 15 (19.3) | 6 (15.7) | 0 (0) | | 21 (16.1) | 22 (16.9) |
| **Medications** | 43 (55.1) | 22 (57.9) | 8 (50.0) | | 73 (55.3) | 70 (53.0) |
| - iron supplement | 16 (23.4) | 10 (26.0) | 5 (31.5) | | 31 (27.6) | 2 (1.6) |
| - analgesia | 11 (14.3) | 5 (18.4) | 2 (12.6) | | 18 (15.8) | 10 (18.1) |
| **Travel abroad in last 3 months** | 12 (15.4) | 6 (15.8) | 4 (25.0) | | 22 (16.7) | 26 (19.7) |
| **Place of delivery** |  |  |  | |  |  |
| - health facility | 77 (98.7) | 34 (89.5) | 16 (100.0) | | 127 (96.2) | 126 (95.5) |
| - home | 1 (1.3) | 4 (10.5) | 0 (0) | | 5 (3.8) | 5 (3.8) |
| **Delivery by caesarian section** | 20 (25.6) | 4 (10.5) | 6 (37.5) | | 30 (22.7) | 37 (28.0) |
| **Initial mode of feeding** |  |  |  | |  |  |
| - exclusively breast fed | 38 (48.7) | 17 (44.7) | 10 (62.5) | | 65 (49.2) | 58 (43.9) |
| - duration of exclusive breast feeding (weeks) median (IQR) | 24.0 (12.0-26.0) | 17.5 (11.0-24.5) | 12.0 (6.0-18.0) | | 20.0 (10.0-26.0) | 24.0 (16.0-26.0) |
| - exclusively bottle fed | 29 (37.2) | 12 (31.6) | 3 (18.8) | | 44 (33.3) | 47 (35.6) |
| - mixed (breast and bottle) | 11 (14.1) | 9 (23.7) | 3 (18.8) | | 23 (17.4) | 27 (20.5) |
| **Age (m) complementary foods first introduced**  median (IQR)^3^ | 6.0 (4.0-6.0) | 5.0 (4.0-6.0) | 5.0 (4.0-6.0) | | 6.0 (4.0-6.0) | 5.0 (4.0-6.0) |

**Notes**

1. Values are n (%) unless stated otherwise
2. Smoking was not known for one participant
3. Age at weaning was not known in 3 participants

CD=Crohn’s disease, UC=ulcerative colitis, IBD-U=inflammatory bowel disease unclassified

**STable 3:** **Demographic characteristics of IBD cases and non-IBD controls according to hospital site.**

| **Variable** | **Hospital site** | | | **Total**  N= 264 (100%) |
| --- | --- | --- | --- | --- |
|  | **Alder Hey**  N=124 (46.9%) | **Bristol**  N=69 (26.1%) | **Birmingham**  N=71 (26.8%) |  |
| **Age (y)**  Median (IQR)  Range | 13.6 (10.5-15.0)  3.7-16.8 | 11.7 (9.6-14.2)  4.4-16.2 | 12.8 (9.6-14.7)  5.9-16.5 | 13.2 (10.2-14.7)  3.7-16.8 |
| **Female** n (%) | 46 (37.1) | 28 (40.6) | 28 (39.4) | 102 (38.6) |
| **Ethnicity** n (%) |  |  |  |  |
| - White | 113 (91.1) | 60 (87.0%) | 41 (57.7) | 214 (81.1) |
| - Asian | 7 (5.6) | 4 (5.8) | 24 (33.8) | 35 (13.3) |
| - Black | 2 (1.6) | 2 (2.9) | 3 (4.2) | 7 (2.7) |
| - Mixed | 2 (1.6) | 3 (4.3) | 2 (2.8) | 7 (2.7) |
| - Other | 0 (0) | 0 (0) | 1 (1.4) | 1 (0.4) |

IBD = inflammatory bowel disease

**STable 4: Disease severity and distribution in IBD**

| **IBD sub-type** | **Baseline** | **Follow-up^1^** | **Baseline** |  |
| --- | --- | --- | --- | --- |
| **Crohn’s disease** | | | | |
| **Severity^2^** | N=78  N (%) | N=53  N (%) | **Distribution^3^** | N=78  N (%) |
| - remission | 6 (7.7) | 24 (45.3) | - distal 1/3 ileum with or without limited caecal disease (L1) | 17 (21.8) |
| - mild | 26 (33.3) | 25 (47.2) | - colonic disease (L2) | 20 (25.6) |
| - moderate | 22 (28.2) | 2 (3.8) | - ileocolonic disease L3 | 41 (52.6) |
| - severe | 24 (30.8) | 2 (3.8) |  |  |
| **Ulcerative colitis** | | | | |
| **Severity^4^** | N=38  N (%) | N=27  N (%) | **Distribution^3^** | N=38  N (%) |
| - remission | 0 (0.0) | 10 (37.0) | - ulcerative proctitis (E1) | 11 (28.9) |
| - mild | 11 (28.9) | 14 (51.9) | - left-sided inflammation (E2) | 2 (5.3) |
| - moderate | 17 (44.7) | 3 (11.1) | - extensive colitis (E3) | 1 (2.6) |
| - severe | 10 (26.3) | 0 (0.0) | - pancolitis (E4) | 24 (63.2) |
| **IBD-unclassified** | | | | |
| - **Severity^4^** | N=16  N (%) | N= 11  N (%) | - **Distribution^3^** | N=16  N (%) |
| - remission | 0 (0.0) | 4 (36.4) | - ulcerative proctitis (E1) | 1 (6.3) |
| - mild | 4 (25.0) | 7 (63.6) | - left-sided inflammation (E2) | 1 (6.3) |
| - moderate | 11 (68.8) | 0 (0.0) | - extensive colitis (E3) | 4 (25.0) |
| - severe | 1 (6.3) | 0 (0.0) | - pancolitis (E4) | 10 (62.5) |

1. Limited to children who provided a stool sample at follow-up
2. Weighted Pediatric Crohn’s Disease Activity Index: ≤12.5 in the absence of corticosteroid treatment, 12.5‐40, 42.5‐57.5 and >57.5 indicated clinical remission, mild, moderate, and severe disease respectively^[[6]](#footnote-6)^
3. Paris classification^[[7]](#footnote-7)^
4. Pediatric Ulcerative Colitis Activity Index (PUCAI), <10 in the absence of corticosteroid treatment, 10-34, 35-64, 65 or above indicated clinical remission, mild, moderate, and severe disease respectively (2)

**STable 5: Compound type of faecal volatile organic compounds identified in IBD cases**

| Ketones or Diketones* | Fatty Acids | Aldehydes or Methylate Aldehydes* | Aromatic compounds |
| --- | --- | --- | --- |
| Butan-2-one  Pentan-2-one  Hexan-2-one  Octan-2-one  Nona n-2-one  Propan-2-one  3-methylbut-3-en-2-one  5 methylheptan-2-one  Pentane-2,3-dione  2 methyl-pent 1en 3 one  Butane-2,3-dione*  Heptan-2-one*  6 methyl-hept-5-en-2-one* | **Short chain**  Acetic acid  Propanoic acid  Butanoic acid  2 methyl-propanoic acid  Pentanoic acid  Propyl acetate  **Branched Chain**  2-methylbutanoic acid  3-methylbutanoic acid  4-methylpentanoic acid  **Straight Chain**  Hexanoic acid  Heptanoic acid | Hexanal  Heptanal  3-methylsulfanylpropanal  Benzaldehyde  Octanal  2-phenylacetaldehyde  Nonanal  Benzaldehyde  3-methylbutanal*  2-methylbutanal*  2-methyl-propanal* | Ethylbenzene  1,4 xylene  Phenol  4 methyl phenol  1 H indole  3 methyl-1-H-indole  4 ethylphenol |
| Alcohols | **Terpenes/ Terpenoids** | **Ester** | **Organosulfur compounds** |
| Butan-1-ol  Pentan-1-ol  Hexan-1-ol  Oct-1-en-3-ol  Ethanol  Propan-1-ol | Gamma-terpinene  D-Limonene  Alpha-pinene  Linalool  Cyclohexa-1,4 diene | Ethyl butanoate  Propyl-propanoate  Butyl butanoate | Methyl-di-sulfanyl-methane  Methyl-tri-sulfanyl-methane |
| Sesquiterpene |  | **Nitrogenous compounds** |  |
| alpha-cubebene  β-Caryophyllene |  | Oxime-, methoxy-phenyl-  (Methyl (Z)- N-hydroxybenzenecarboximidate) |  |

**Source: https://pubchem.ncbi.nlm.nih.gov/**

**STable 6: Number of VOCs according to the recruitment site**

| **Site** | **Mean** | **Median (SD)** | **SEM** | **CV** | **P value^1^** | **Comparison** | **P value (adjusted)^2^** |
| --- | --- | --- | --- | --- | --- | --- | --- |
| **Birmingham** | 39.08 | 39 (9.89) | 1.17 | 0.25 | 0.02 | Bristol- Birmingham | 0.555 |
| **Bristol** | 39.17 | 40 (11.51) | 1.39 | 0.29 |  | Liverpool- Bristol | 0.034 |
| **Liverpool** | 36.08 | 37(9.96) | 0.89 | 0.28 |  | Liverpool- Birmingham | 0.079 |

**Note:**

1. Kruskal-Wallis test
2. Benjamini-Hochberg correction

SEM=standard error of the mean; CV=coefficient of variation

**STable 7: Difference in faecal volatile organic compound abundance in the Crohn’s disease/matched controls pairs**

| **Retention time and volatile organic compound** | **Median (IQR) difference^1^** | **P-Value^2^** |
| --- | --- | --- |
| **Ketone or diketone*** | | |
| - 10:50 Butan-2-one | - 0.57 (-1.85- 0.30) | **0.001** |
| - 10:13 Butane-2,3-dione* | 0.00 (-1.50- 0.77) | 0.139 |
| - 13:73 Pentan-2-one | -0.011 (-1.39- 0.44) | 0.07 |
| - 14: 06 Pentane-2,3-dione | - 0.12 (-1.73- 1.48) | 0.52 |
| - 17:98 Hexan-2-one | 0.00 (- 1.79- 0.59) | 0.089 |
| - 21:86 Heptan-2-one* | - 0.19 (-1.59- 0.31) | **0.044** |
| - 25:56 Octan-2-one | 0.00 (- 0.87- 0.27) | 0.119 |
| - 29:00 Nonan-2-one | 0.00 (-0.59- 0.24) | 0.700 |
| - 7:37 Propan-2-one | 0.00 (-0.96- 1.02) | 0.892 |
| - 16:84 2-Methylpent-1-en-3-one | 0.00 (-1.66- 0.76) | 0.231 |
| - 13:08 3-Methylbut-3-en-2-one | 0.00 (- 0.52- 0.23) | 0.059 |
| - 24:71 5-Methylheptan-2-one | 0.00 (-1.25- 1.96) | 0.771 |
| - 25:46 6-methylhept-5-en-2-one* | -0.16 (-1.68- 0.81) | 0.157 |
| **Fatty Acids – short-chain; saturated** | | |
| - 12:43 Acetic acid | 0.007 (-3.07- 2.27) | 0.419 |
| - 19:45 Butanoic acid | -0.12 (-0.99- 0.83) | 0.456 |
| - 16:03 Propanoic acid | 0.020 (-1.30- 1.01) | 0.634 |
| **Fatty Acids – branched-chain; saturated**^$^ | | |
| - 18:15 2-Methylpropanoic acid | -0.13 (-1.56- 1.04) | 0.288 |
| - 21:38 3-Methylbutanoic acid^$^ | -0.14 (-0.89- 1.45) | 0.852 |
| - 21:73 2-Methylbutanoic acid | - 0.14 (-1.27- 1.24) | 0.638 |
| - 25:25 4-Methylpentanoic acid | 0.42 (-1.95- 2.45) | 0.170 |
| **Fatty Acids – medium-chain; saturated** | | |
| - 22:94 Pentanoic acid | -0.48 (-1.52- 1.07) | 0.107 |
| - 26:25 Hexanoic acid | -0.52 (-2.98- 1.31) | **0.024** |
| - 29:20 Hepatanoic acid | 0.00 (- 0.68- 0.04) | 0.210 |
| **Mono carboxylic acid** | | |
| - 31:48 Cyclohexanecarboxylic acid | 0.00 (- 0.20-1.46) | 0.218 |
| **Aldehydes; methylate aldehydes**^α^**; alpha hydrogen**^β^**; aromatic aldehyde**^γ^ | | |
| - 12:47 3-methylbutanal^α^ | - 0.17 (-1.89- 1.59) | 0.463 |
| - 12:72 2-methylbutanal^α^ | - 0.00 (-1.51- 1.29) | 0.440 |
| - 18:21 Hexanal | 0.00 (-1.49- 1.84) | 0.982 |
| - 22:15 Heptanal | - 0.07 (-1.19- 0.74) | 0.285 |
| - 23:39 3-methylsulfanylpropanal^β^ | - 0.79 (-2.34- 0.25) | **<0.001** |
| - 25:38 Benzaldehyde^γ^ | 0.12 (-0.96- 1.25) | 0.252 |
| - 25:88 Octanal | -0.31 (-1.83- 0.41) | **0.020** |
| - 28:53 2-phenylacetaldehyde | -0.41 (-1.19- 0.46) | **0.006** |
| - 29:39 Nonanal | 0.00 (-1.67- 0.07) | 0.217 |
| - 9:00 2-methylpropanal^α^ | 0.00 (- 0.89- 0.60) | 0.523 |
| **Aromatic compounds; aromatic hydrocarbon**^@^**; aromatic heterocyclic**^&^ | | |
| - 20:23 Ethylbenzene^@^ | 0.00 (-0.29- 0.29) | 0.766 |
| - 20:53 1,4-xylene | 0.00 (- 0.34- 0.34) | 0.279 |
| - 27:90 Phenol | 1.97 (-0.46- 3.91) | **<0.001** |
| - 30:78 4-methylphenol | -0.14 (-2.40- 1.62) | 0.178 |
| - 33:63 4-ethylphenol | 0.00 (-0.09- 0.09) | 0.403 |
| - 38:39 1H-indole^&^ | 0.38 (-1.32- 1.82) | 0.118 |
| - 41:12 3-methyl-1h-indole^&^ | - 0.02 (-1.97- 1.21) | 0.363 |
| **Alcohols;** **primary**^θ^**; secondary**^ρ^ | | |
| - 17:26 Pentan-1-ol^θ^ | 0.00 (-1.66- 0.75) | 0.414 |
| - 21:21 Hexan-1-ol^ρ^ | 0.00 (-0.34- 0.34) | 0.323 |
| - 13:28 Butan-1-ol^θ^ | 0.00 (-1.48- 2.54) | 0.470 |
| - 25:10 Oct-1-en-3-ol^ρ^ | 0.00 (0.00- 0.45) | **0.001** |
| - 6:65 Ethanol^θ^ | 0.00 (0.00- 0.79) | **0.002** |
| - 9:54 Propan-1-ol^θ^ | 0.81 (-1.10- 2.79) | **<0.001** |
| **Organosulfur compounds** | | |
| - 15:92 (Methyldisulfanyl) methane | - 0.70 (-2.91- 1.36) | **0.040** |
| - 25:22 (Methyltrisulfanyl) methane | 0.00 (-2.19- 2.42) | 0.714 |
| **Monoterpenes or Terpenoids**^®^ | | |
| - 22:55 Alpha-pinene^3^ | 0.00 (-1.44- 0.11) | 0.172 |
| - 26:10 D-Limonene^4^ | - 0.99 (-2.34- 0.52) | **<0.001** |
| - 26:91 Gamma-terpinene^5^ | 0.00 (-1.51- 1.43) | 0.729 |
| - 28:98 Linalool^6^ | 0.00 (-0.28- 0.24) | 0.175 |
| - 30:81 Cyclohexa-1,4-diene^®^ | 0.00 (-0.30- 0.30) | 0.627 |
| **Ester** | | |
| - 14:28 Propyl acetate | 0.00 (-0.31- 0.31) | 0.969 |
| - 24:69 Butyl butanoate | 0.00 (-1.21- 1.37) | 0.982 |
| - 17:93 Propyl propanoate | 0.00 (-1.22- 0.00) | 0.450 |
| - 17:55 Ethyl butanoate | 0.00 (-0.25- 1.46) | 0.589 |
| **Nitrogenous compounds** | | |
| - 23:86 Oxime-, methoxy-phenyl-^7^ | 0.00 (-0.37- 0.28) | 0.613 |
| **Sesquiterpene; tricyclic**^π^ **or primary**^Ω^ | | |
| - 37:56 Alpha-Cubebene^8π^ | 0.00 (-1.33- 0.09) | **0.047** |
| - 39:51 β-Caryophyllene^9Ω^ | -0.76 (-2.19- 0.73) | **0.026** |
| **Unclassified** | | |
| - 24:31 (1S)-6,6-dimethyl-4-methylidenebicyclo [3.1.1] heptane | -0.09 (-2.31- 0.73) | 0.052 |

**Notes:**

1. A negative value occurs when VOCs abundance is lower in cases than controls and *vice versa*
2. Wilcoxon Signed Rank Test
3. 2,6,6-trimethyl-bicyclo 3.1.1 hept-2-ene
4. (4R)-1-methyl-4-prop-1-en-2-ylcyclohexene
5. 1-methyl-4-propan-2-ylcyclohexa-1,4-diene
6. 3,7-dimethylocta-1,6-dien-3-ol
7. Methyl-ZN-hydroxy-benzene-carboximidate
8. (1R,5S,6R,7S,10R)-4,10-dimethyl-7-propan-2-yltricyclo [4.4.0.01,5] dec-3 ene_ (alpha-Cubebene
9. (1R,4E,9S)-4,11,11-trimethyl-8-methylidenebicyclo [7.2.0] undec-4-ene_(Caryophyllene)

Statistically-significant differences are shown in bold type

**STable 8: Faecal volatile organic compounds abundance according to disease severity in Crohn’s disease**

| **Retention time and faecal volatile organic compound** | **Mild**  **Mean (SD)^1^** | **Moderate Mean (SD)** | **Severe**  **Mean (SD)** | **HSD^2^ Moderate-Mild** | **HSD Severe-Mild** | **HSD Severe-Moderate** | **P-Value^3^** |
| --- | --- | --- | --- | --- | --- | --- | --- |
| **Ketones or diketones*** | | | | | | | |
| - 10:50 Butan-2-one | 14.23 (1.23) | 14.54 (1.60) | 13.86 (1.52) | 0.718 | 0.607 | 0.250 | 0.280 |
| - 10:13 Butane-2,3-dione* | 14.38 (1.20) | 14.54 (1.01) | 14.20 (1.33) | 0.879 | 0.856 | 0.617 | 0.644 |
| - 13:73 Pentan-2-one | 14.47 (1.40) | 14.25 (1.48) | 14.35 (1.44) | 0.847 | 0.947 | 0.972 | 0.856 |
| - 14: 06 Pentane-2,3-dione | 14.61 (1.53) | 15.21 (1.54) | 14.95 (1.62) | 0.349 | 0.693 | 0.841 | 0.371 |
| - 17:98 Hexan-2-one | 13.50 (1.28) | 13.51 (1.28) | 13.49 (1.65) | 1.000 | 1.000 | 0.999 | 0.999 |
| - 21:86 Heptan-2-one* | 14.55 (1.32) | 14.41 (1.48) | 14.11 (1.24) | 0.916 | 0.449 | 0.743 | 0.479 |
| - 25:56 Octan-2-one | 13.44 (1.08) | 13.33 (1.12) | 13.34 (0.94) | 0.921 | 0.924 | 1.000 | 0.902 |
| - 29:00 Nonan-2-one | 13.09 (1.14) | 13.20 (1.31) | 13.05 (1.21) | 0.942 | 0.991 | 0.907 | 0.909 |
| - 7:37 Propan-2-one (acetone) | 14.32 (1.01) | 14.87 (0.99) | 14.34 (1.20) | 0.161 | 0.998 | 0.222 | 0.141 |
| - 16:84 2-Methylpent-1-en-3-one | 12.30 (1.46) | 12.78 (1.63) | 12.38 (1.51) | 0.488 | 0.976 | 0.653 | 0.499 |
| - 13:08 3-Methylbut-3-en-2-one | 14.09 (0.63) | 14.08 (0.70) | 14.00 (0.50) | 0.997 | 0.859 | 0.910 | 0.860 |
| - 24:71 5-Methylheptan-2-one | 13.47 (1.41) | 13.73 (1.53) | 13.70 (1.54) | 0.793 | 0.833 | 0.996 | 0.766 |
| - 25:46 6-methylhept-5-en-2-one* | 15.10 (1.34) | 15.71 (1.53) | 14.50 (1.67) | 0.310 | 0.310 | **0.021** | **0.029** |
| **Fatty Acids – short-chain; saturated** | | | | | | | |
| - 12.43 Acetic acid | 16.93 (2.16) | 16.70 (2.33) | 16.88 (2.26) | 0.925 | 0.995 | 0.961 | 0.929 |
| - 19:45 Butanoic acid | 19.96 (1.45) | 19.62 (0.99) | 18.89 (1.84) | 0.686 | **0.023** | 0.216 | **0.030** |
| - 16:03 Propanoic acid | 17.80 (1.43) | 17.93 (1.54) | 17.23 (2.03) | 0.955 | 0.419 | 0.331 | 0.305 |
| **Fatty Acids – branched chain; saturated**^$^ | | | | | | | |
| - 18:15 2-Methylpropanoic acid | 17.22 (1.71) | 17.67 (1.25) | 16.11 (2.49) | 0.669 | 0.081 | **0.018** | **0.017** |
| - 21:38 3-Methylbutanoic acid^$^ | 18.96 (1.15) | 18.91 (1.11) | 17.53 (2.19) | 0.994 | **0.003** | **0.009** | **0.002** |
| - 21:73 2-Methylbutanoic acid | 18.59 (1.19) | 18.61 (1.16) | 17.17 (2.30) | 0.999 | **0.005** | **0.009** | **0.002** |
| - 25:25 4-Methylpentanoic acid | 16.24 (1.68) | 15.31 (2.35) | 14.88 (2.36) | 0.260 | 0.050 | 0.761 | 0.052 |
| **Fatty Acids – medium-chain; saturated** | | | | | | | |
| - 22:94 Pentanoic acid | 19.22 (1.50) | 18.71 (1.36) | 17.70 (2.31) | 0.551 | **0.006** | 0.131 | **0.008** |
| - 26:25 Hexanoic acid | 17.20 (2.08) | 16.72 (1.98) | 15.57 (2.31) | 0.697 | **0.016** | 0.166 | **0.020** |
| - 29:20 Hepatanoic acid | 15.13 (1.47) | 14.59 (0.78) | 14.64 (0.68) | 0.186 | 0.234 | 0.986 | 0.134 |
| **Mono Carboxylic acid** | | | | | | | |
| - 31:48 Cyclohexanecarboxylic acid | 14.26 (1.82) | 13.67 (1.52) | 13.72 (1.54) | 0.410 | 0.443 | 0.996 | 0.336 |
| **Aldehydes; methylate Aldehydes**^α^**; alpha hydrogen**^β^**;**  **aromatic aldehyde**^γ^ | | | | | | | |
| - 12:47 3-methylbutanal^α^ | 14.71 (1.84) | 15.37 (1.61) | 15.03 (2.14) | 0.409 | 0.794 | 0.814 | 0.440 |
| - 12:72 2-methylbutanal^α^ | 14.66 (1.69) | 14.89 (1.85) | 14.94 (1.71) | 0.877 | 0.817 | 0.995 | 0.805 |
| - 18:21 Hexanal | 13.24 (1.43) | 13.00 (1.55) | 13.51 (1.57) | 0.844 | 0.775 | 0.490 | 0.520 |
| - 22:15 Heptanal | 13.15 (0.89) | 13.29 (1.03) | 13.22 (1.30) | 0.884 | 0.973 | 0.969 | 0.894 |
| - 23:39 3-methylsulfanylpropanal^β^ | 13.10 (1.24) | 13.56 (1.21) | 12.72 (1.39) | 0.393 | 0.530 | 0.075 | 0.092 |
| - 25:38 Benzaldehyde^γ^ | 15.52 (0.84) | 15.97 (1.46) | 15.85 (1.71) | 0.452 | 0.626 | 0.956 | 0.437 |
| - 25:88 Octanal | 13.18 (1.30) | 13.37 (1.37) | 13.17 (1.59) | 0.880 | 1.000 | 0.889 | 0.866 |
| - 28:53 2-phenylacetaldehyde | 16.70 (0.83) | 16.68 (0.99) | 16.21 (1.70) | 0.998 | 0.288 | 0.382 | 0.265 |
| - 29:39 Nonanal | 12.72 (1.05) | 12.73 (1.17) | 12.93 (1.36) | 0.998 | 0.790 | 0.849 | 0.785 |
| - 9:00 2-methylpropanal^α^ | 12.97 (0.63) | 13.39 (0.83) | 13.58 (1.26) | 0.227 | 0.045 | 0.779 | 0.045 |
| **Aromatic compounds; aromatic hydrocarbon**^@^**; aromatic heterocyclic**^&^ | | | | | | | |
| - 20:23 Ethylbenzene^@^ | 12.24 (0.94) | 11.82 (0.64) | 12.22 (1.17) | 0.252 | 0.998 | 0.323 | 0.229 |
| - 20:53 1,4-xylene | 12.10 (0.66) | 11.98 (0.69) | 12.55 (1.26) | 0.885 | 0.157 | 0.088 | 0.075 |
| - 27:90 Phenol | 18.07 (1.90) | 17.95 (2.36) | 17.76 (3.52) | 0.985 | 0.901 | 0.967 | 0.909 |
| - 30:78 4-methylphenol | 19.80 (1.69) | 19.32 (1.88) | 18.70 (2.15) | 0.635 | 0.087 | 0.510 | 0.107 |
| - 33:63 4-ethylphenol | 13.95 (0.41) | 14.20 (0.88) | 14.07 (0.61) | 0.316 | 0.742 | 0.767 | 0.346 |
| - 38:39 1H-indole^&^ | 18.51 (1.27) | 18.39 (1.71) | 18.57 (1.80) | 0.957 | 0.991 | 0.922 | 0.926 |
| - 41:12 3-methyl-1h-indole^&^ | 14.87 (1.78) | 15.28 (1.73) | 14.84 (1.35) | 0.642 | 0.998 | 0.644 | 0.597 |
| **Alcohols;** **primary**^θ^**; secondary**^ρ^ | | | | | | | |
| - 17:26 Pentan-1-ol^θ^ | 12.62 (1.44) | 12.99 (1.77) | 12.43 (1.51) | 0.673 | 0.897 | 0.455 | 0.477 |
| - 21:21 Hexan-1-ol^ρ^ | 12.65 (1.61) | 12.18 (0.55) | 12.67 (1.51) | 0.434 | 0.999 | 0.454 | 0.387 |
| - 13:28 Butan-1-ol^θ^ | 13.89 (1.72) | 13.72 (1.54) | 13.77 (1.78) | 0.931 | 0.966 | 0.993 | 0.931 |
| - 25:10 Oct-1-en-3-ol^ρ^ | 12.65 (0.66) | 12.91 (0.74) | 13.10 (1.11) | 0.489 | 0.121 | 0.735 | 0.136 |
| - 6:65 Ethanol^θ^ | 15.76 (0.59) | 15.84 (0.81) | 15.80 (0.69) | 0.919 | 0.976 | 0.983 | 0.925 |
| - 9:54 Propan-1-ol^θ^ | 15.19 (1.72) | 15.21 (1.75) | 15.42 (1.89) | 0.998 | 0.874 | 0.915 | 0.873 |
| **Organosulfur compounds** | | | | | | | |
| - 15:92 (Methyldisulfanyl) methane | 13.96 (1.70) | 14.45 (2.20) | 13.80 (1.79) | 0.615 | 0.942 | 0.465 | 0.470 |
| - 25:22 (Methyltrisulfanyl) methane | 13.79 (1.72) | 13.97 (2.06) | 14.11(1.77) | 0.931 | 0.792 | 0.964 | 0.806 |
| **Monoterpenes or Terpenoids**^®^ | | | | | | | |
| - 22:55 Alpha-pinene^4^ | 12.94 (1.09) | 13.06 (1.21) | 12.82 (0.97) | 0.914 | 0.914 | 0.735 | 0.756 |
| - 26:10 D-Limonene^5^ | 15.24 (1.34) | 15.03 (1.69) | 14.11 (1.53) | 0.867 | **0.018** | 0.102 | **0.019** |
| - 26:91 Gamma-terpinene^6^ | 13.31 (2.19) | 12.70 (1.33) | 12.57 (1.70) | 0.456 | 0.292 | 0.965 | 0.269 |
| - 28:98 Linalool^7^ | 12.31 (1.15) | 12.01 (0.83) | 11.95 (0.92) | 0.506 | 0.379 | 0.983 | 0.343 |
| - 30:81 Cyclohexa-1,4-diene^®^ | 18.92 (1.07) | 18.83 (1.12) | 18.64 (1.09) | 0.962 | 0.612 | 0.811 | 0.634 |
| **Ester** | | | | | | | |
| - 14:28 Propyl acetate | 13.24 (1.51) | 12.69 (0.93) | 13.00 (1.10) | 0.252 | 0.755 | 0.676 | 0.284 |
| - 24:69 Butyl butanoate | 14.10 (1.76) | 13.14 (0.82) | 13.26 (1.35) | 0.045 | 0.080 | 0.956 | **0.027** |
| - 17:93 Propyl propanoate | 12.37 (1.38) | 12.42 (1.27) | 12.36 (1.04) | 0.985 | 1.000 | 0.982 | 0.981 |
| - 17:55 Ethyl butanoate | 13.84 (1.82) | 13.07 (1.06) | 13.17 (1.17) | 0.144 | 0.214 | 0.969 | 0.107 |
| **Nitrogenous compounds** | | | | | | | |
| - 23:86 Oxime-, methoxy-phenyl-^8^ | 15.45 (0.59) | 15.61 (0.68) | 16.08 (1.32) | 0.803 | 0.030 | 0.181 | **0.035** |
| **Sesquiterpene; tricyclic**^π^ **or primary**^Ω^ | | | | | | | |
| - 37:56 Alpha-Cubebene^9π^ | 12.37 (1.25) | 12.30 (1.33) | 11.87 (0.89) | 0.981 | 0.270 | 0.427 | 0.267 |
| - 39:51 β-Caryophyllene^10Ω^ | 13.99 (1.57) | 14.04 (1.64) | 13.06 (1.38) | 0.992 | 0.070 | 0.083 | 0.046 |
| **Unclassified** | | | | | | | |
| - 24:31 (1S)-6,6-dimethyl-4 methylidenebicyclo [3.1.1] heptane | 13.95 (1.73) | 13.52 (1.51) | 13.14 (1.38) | 0.578 | 0.142 | 0.698 | 0.164 |

**Notes:**

1. Mild includes disease classified as in remission at baseline assessment
2. HSD: Tukey's honestly significant difference test
3. One-way ANOVA
4. 2,6,6-trimethyl-bicyclo 3.1.1 hept-2-ene
5. (4R)-1-methyl-4-prop-1-en-2-ylcyclohexene
6. 1-methyl-4-propan-2-ylcyclohexa-1,4-diene
7. 3,7-dimethylocta-1,6-dien-3-ol
8. Methyl-ZN-hydroxy-benzene-carboximidate
9. (1R,5S,6R,7S,10R)-4,10-dimethyl-7-propan-2-yltricyclo [4.4.0.01,5] dec-3-ene_ (alpha Cubebene)
10. (1R,4E,9S)-4,11,11-trimethyl-8-methylidenebicyclo [7.2.0] undec-4-ene_(Caryophyllene)

Statistically-significant differences are shown in bold type

**STable 9: Associations between VOCs abundance and FC at baseline in children subsequently diagnosed with IBD^1^**

| **Retention time and faecal volatile organic compound** | **CD only** | **Colitis only** | **Controls only** |
| --- | --- | --- | --- |
| **Intercept** | 1423.516667 | 2505.963129 | 110.7146212 |
| **Ketones or diketones*** | | | |
| - 10:50 Butan-2-one | 0 | 0 | 0 |
| - 10:13 Butane-2,3-dione* | 0 | 0 | 0 |
| - 13:73 Pentan-2-one | 0 | 0 | 0 |
| - 14: 06 Pentane-2,3-dione | 0 | 0 | 0 |
| - 17:98 Hexan-2-one | 0 | 0 | 0 |
| - 21:86 Heptan-2-one* | 0 | 0 | 0 |
| - 25:56 Octan-2-one | 0 | 0 | 0 |
| - 29:00 Nonan-2-one | 0 | 0 | 0 |
| - 7:37 Propan-2-one (acetone) | 0 | 0 | 0 |
| - 16:84 2-Methylpent-1-en-3-one | 0 | 0 | 0 |
| - 13:08 3-Methylbut-3-en-2-one | 0 | 0 | 0 |
| - 24:71 5-Methylheptan-2-one | 0 | 0 | 0 |
| - 25:46 6-methylhept-5-en-2-one* | 0 | -99.80 | 0 |
| **Fatty Acids – short chain; saturated** | | | |
| - 12:43 Acetic acid | 0 | 0 | 0 |
| - 19:45 Butanoic acid | 0 | 0 | 0 |
| - 16:03 Propanoic acid | 0 | 0 | 0 |
| **Fatty Acids – branched-chain; saturated**^$^ | | | |
| - 18:15 2-Methylpropanoic acid | 0 | 0 | 0 |
| - 21:38 3-Methylbutanoic acid^$^ | 0 | 0 | 0 |
| - 21:73 2-Methylbutanoic acid |  |  |  |
| - 25:25 4-Methylpentanoic acid | 0 | 0 | 0 |
| **Fatty Acids – medium-chain; saturated** | | | |
| - 22:94 Pentanoic acid | 0 | 0 | 0 |
| - 26:25 Hexanoic acid | 0 | 0 | 0 |
| - 29:20 Hepatanoic acid | 0 | 0 | 0 |
| **Monocarboxylic acid** | | | |
| - 31:48 Cyclohexanecarboxylic acid | 0 | 0 | 0 |
| **Aldehydes; methylate aldehydes**^α^**; alpha hydrogen**^β^**; aromatic aldehyde**^γ^ | | | |
| - 12:47 3-methylbutanal^α^ | 0 | 0 | 0 |
| - 12:72 2-methylbutanal^α^ | 0 | 0 | 0 |
| - 18:21 Hexanal | 0 | 0 | 0 |
| - 22:15 Heptanal | 0 | 0 | 0 |
| - 23:39 3-methylsulfanylpropanal^β^ | 0 | 0 | 0 |
| - 25:38 Benzaldehyde^γ^ | 0 | 0 | 0 |
| - 25:88 Octanal | 0 | 0 | 0 |
| - 28:53 2-phenylacetaldehyde | 0 | 0 | 0 |
| - 29:39 Nonanal | 0 | 0 | 0 |
| - 9:00 2-methylpropanal^α^ | 0 | 0 | 0 |
| **Aromatic compounds; aromatic hydrocarbon**^@^**; aromatic heterocyclic**^&^ | | | |
| - 20:23 Ethylbenzene^@^ | 0 | 0 | 0 |
| - 20:53 1,4-xylene | 0 | 0 | 0 |
| - 27:90 Phenol | 8.02 | 0 | 0 |
| - 30:78 4-methylphenol | 0 | 0 | 0 |
| - 33:63 4-ethylphenol | 0 | 0 | 0 |
| - 38:39 1H-indole^&^ | 0 | 0 | 0 |
| - 41:12 3-methyl-1h-indole^&^ | 0 | 0 | 0 |
| **Alcohols;** **primary**^θ^**; secondary**^ρ^ | | | |
| - 17:26 Pentan-1-ol^θ^ | 0 | 0 | 0 |
| - 21:21 Hexan-1-ol^ρ^ | 0 | 0 | 0 |
| - 13:28 Butan-1-ol^θ^ | 0 | 0 | 0 |
| - 25:10 Oct-1-en-3-ol^ρ^ | 0 | 0 | 0 |
| - 6:65 Ethanol^θ^ | 0 | 20.69 | 0 |
| - 9:54 Propan-1-ol^θ^ | 0 | 0 | 0 |
| **Organosulfur compounds** | | | |
| - 15:92 (Methyldisulfanyl) methane | 0 | 0 | 0 |
| - 25:22 (Methyltrisulfanyl) methane | 0 | 0 | 0 |
| **Monoterpenes or Terpenoids**^®^ | | | |
| - 22:55 Alpha-pinene^2^ | 0 | 0 | 0 |
| - 26:10 D-Limonene^3^ | 0 | 0 | 0 |
| - 26:91 Gamma-terpinene^4^ | 0 | 0 | 0 |
| - 28:98 Linalool^5^ | 0 | 0 | 0 |
| - 30:81 Cyclohexa-1,4-diene^®^ | 0 | 0 | 0 |
| **Ester** | | | |
| - 14:28 Propyl acetate | 0 | 0 | 0 |
| - 24:69 Butyl butanoate | 0 | 0 | 0 |
| - 17:93 Propyl propanoate | 0 | 0 | 0 |
| - 17:55 Ethyl butanoate | 0 | 0 | 0 |
| **Nitrogenous compounds** | | | |
| - 23:86 Oxime-, methoxy-phenyl-^6^ | 0 | 0 | 0 |
| **Sesquiterpene; tricyclic**^π^ **or primary**^Ω^ | | | |
| - 37:56 Alpha-Cubebene^7π^ | 0 | 0 | 0 |
| - 39:51 β-Caryophyllene^8Ω^ | 0 | 0 | 0 |
| **Unclassified** |  |  |  |
| - 24:31(1S)-6,6-dimethyl-4-methylidenebicyclo [3.1.1] heptane | 0 | 0 | 0 |

**Notes:**

1. Performed by LASSO regression; l1-panelty constraint forces non-significant beta coefficients to go to zero leaving only those that are changing with respect to the parameter of interest
2. 2,6,6-trimethyl-bicyclo 3.1.1 hept-2-ene
3. (4R)-1-methyl-4-prop-1-en-2-ylcyclohexene
4. 1-methyl-4-propan-2-ylcyclohexa-1,4-diene
5. 3,7-dimethylocta-1,6-dien-3-ol
6. Methyl-ZN-hydroxy-benzene-carboximidate
7. (1R,5S,6R,7S,10R)-4,10-dimethyl-7-propan-2-yltricyclo [4.4.0.01,5] dec-3-ene_(alpha-Cubebene)
8. (1R,4E,9S)-4,11,11-trimethyl-8-methylidenebicyclo [7.2.0] undec-4-ene_(Caryophylle)

**STable 10: Faecal volatile organic compound abundance according to disease distribution in Crohn’s disease**

| **Retention time and faecal volatile organic compound** | **L1**  **N=17**  **Mean (SD)** | **L2**  **N=20**  **Mean (SD)** | **L3**  **N=41**  **Mean (SD)** | **HSD^1^**  **L1-L2** | **HSD**  **L3-L1** | **HSD**  **L3-L2** | **P-Value^2^** |
| --- | --- | --- | --- | --- | --- | --- | --- |
| **Ketones or diketones*** | | | | | | | |
| - 10:50 Butan-2-one | 14.85 (1.23) | 14.28 (1.42) | 13.90 (1.46) | 0.442 | 0.055 | 0.575 | 0.067 |
| - 10:13 Butane-2,3-dione* | 14.35 (1.13) | 14.71 (1.24) | 14.21 (1.17) | 0.63 | 0.910 | 0.277 | 0.310 |
| - 13:73 Pentan-2-one | 14.67 (1.49) | 14.66 (1.39) | 14.11 (1.38) | 1.000 | 0.348 | 0.320 | 0.219 |
| - 14:06 Pentane-2,3-dione | 15.37 (1.29) | 15.34 (1.55) | 14.46 (1.58) | 0.998 | 0.099 | 0.089 | **0.038** |
| - 17:98 Hexan-2-one | 14.35 (1.63) | 13.78 (1.31) | 13.01 (1.09) | 0.362 | **0.001** | 0.078 | **0.001** |
| - 21:86 Heptan-2-one* | 15.00 (1.12) | 14.35 (1.41) | 14.13 (1.33) | 0.289 | 0.061 | 0.817 | 0.076 |
| - 25:56 Octan-2-one | 13.77 (1.28) | 13.33 (1.02) | 13.24 (0.92) | 0.410 | 0.189 | 0.947 | 0.212 |
| - 29:00 Nonan-2-one | 13.71 (1.52) | 12.91 (1.09) | 12.96 (1.04) | 0.104 | 0.072 | 0.990 | 0.063 |
| - 7:37 Propan-2-one (acetone) | 14.46 (1.13) | 14.70 (1.04) | 14.38 (1.09) | 0.778 | 0.970 | 0.537 | 0.565 |
| - 16:84 2-Methylpent-1-en-3-one | 13.06 (1.55) | 12.37 (1.41) | 12.25 (1.53) | 0.355 | 0.158 | 0.954 | 0.177 |
| - 13:08 3-Methylbut-3-en-2-one | 14.14 (0.64) | 14.13 (0.66) | 13.99 (0.57) | 0.999 | 0.670 | 0.666 | 0.572 |
| - 24:71 5-Methylheptan-2-one | 14.39 (1.41) | 13.29 (1.42) | 13.45 (1.44) | 0.059 | 0.066 | 0.913 | **0.044** |
| - 25:46 6-methylhept-5-en-2-one* | 15.79 (0.77) | 15.09 (1.53) | 14.79 (1.74) | 0.346 | 0.065 | 0.754 | 0.081 |
| **Fatty Acids – short chain; saturated** | | | | | | | |
| - 12:43 Acetic acid | 16.80 (2.05) | 17.01 (2.46) | 16.79 (2.20) | 0.954 | 1.000 | 0.929 | 0.930 |
| - 19:45 Butanoic acid | 19.66 (1.03) | 19.48 (1.77) | 19.51 (1.61) | 0.932 | 0.940 | 0.997 | 0.929 |
| - 16:03 Propanoic acid | 17.99 (1.24) | 17.36 (2.01) | 17.67 (1.66) | 0.491 | 0.781 | 0.781 | 0.491 |
| **Fatty Acids – branched-chain; saturated**^$^ | | | | | | | |
| - 18:15 2-Methylpropanoic acid | 17.61 (1.14) | 17.03 (1.92) | 16.74 (2.22) | 0.646 | 0.282 | 0.851 | 0.314 |
| - 21:38 3-Methylbutanoic acid^$^ | 18.71 (0.85) | 18.59 (1.59) | 18.38 (1.93) | 0.974 | 0.780 | 0.895 | 0.773 |
| - 21:73 2-Methylbutanoic acid | 18.48 (0.97) | 18.08 (1.87) | 18.06 (1.90) | 0.763 | 0.678 | 0.999 | 0.684 |
| - 25:25 4-Methylpentanoic acid | 15.93 (2.20) | 15.61 (2.04) | 15.38 (2.23) | 0.894 | 0.654 | 0.921 | 0.675 |
| **Fatty Acids - medium-chain; saturated** | | | | | | | |
| - 22:94 Pentanoic acid | 19.03 (1.42) | 18.52 (1.97) | 18.47 (1.96) | 0.682 | 0.559 | 0.996 | 0.571 |
| - 26:25 Hexanoic acid | 17.09 (2.21) | 16.96 (2.28) | 16.15 (2.15) | 0.982 | 0.300 | 0.368 | 0.982 |
| - 29:20 Hepatanoic acid | 15.04 (1.24) | 15.14 (1.44) | 14.59 (0.81) | 0.961 | 0.332 | 0.166 | 0.131 |
| **Monocarboxylic acid** | | | | | | | |
| - 31:48 Cyclohexanecarboxylic acid | 14.00 (1.56) | 13.72 (1.56) | 14.00 (1.77) | 0.865 | 1.000 | 0.814 | 0.812 |
| **Aldehydes; methylate aldehydes**^α^**; alpha hydrogen**^β^**; aromatic aldehyde**^γ^**γ** | | | | | | | |
| - 12:47 3-methylbutanal^α^ | 15.79 (1.18) | 14.77 (1.97) | 14.78 (2.00) | 0.223 | 0.146 | 1.000 | 0.142 |
| - 12:72 2-methylbutanal^α^ | 15.46 (1.00) | 14.61 (1.47) | 14.64 (2.02) | 0.294 | 0.228 | 0.998 | 0.216 |
| - 18:21 Hexanal | 13.66 (1.67) | 13.05 (1.51) | 13.19 (1.42) | 0.439 | 0.525 | 0.939 | 0.434 |
| - 22:15 Heptanal | 13.16 (1.02) | 13.26 (1.09) | 13.21 (1.08) | 0.958 | 0.982 | 0.988 | 0.962 |
| - 23:39 3-methylsulfanylpropanal^β^ | 13.72 (1.16) | 12.96 (1.32) | 12.93 (1.31) | 0.182 | 0.093 | 0.996 | 0.095 |
| - 25:38 Benzaldehyde^γ^ | 16.15 (1.36) | 16.02 (1.50) | 15.45 (1.20) | 0.954 | 0.165 | 0.257 | 0.112 |
| - 25:88 Octanal | 13.58 (1.57) | 13.31 (1.34) | 13.05 (1.36) | 0.828 | 0.387 | 0.770 | 0.404 |
| - 28:53 2-phenylacetaldehyde | 17.07 (0.64) | 16.25 (1.48) | 16.47 (1.28) | 0.098 | 0.190 | 0.782 | 0.101 |
| - 29:39 Nonanal | 12.96 (1.32) | 12.65 (1.17) | 12.78 (1.14) | 0.713 | 0.868 | 0.910 | 0.735 |
| - 9:00 2-methylpropanal^α^ | 13.46 (1.20) | 13.21 (0.81) | 13.23 (0.90) | 0.714 | 0.699 | 0.995 | 0.677 |
| **Aromatic compounds; aromatic hydrocarbon**^@^**; aromatic heterocyclic**^&^ | | | | | | | |
| - 20:23 Ethylbenzene^@^ | 12.50 (1.10) | 11.85 (0.83) | 12.08 (0.92) | 0.100 | 0.287 | 0.636 | 0.116 |
| - 20:53 1,4-xylene | 12.23 (0.85) | 12.30 (1.12) | 12.15 (0.84) | 0.968 | 0.955 | 0.821 | 0.832 |
| - 27:90 Phenol | 17.86 (2.68) | 17.74 (2.32) | 18.07 (2.72) | 0.989 | 0.958 | 0.889 | 0.890 |
| - 30:78 4-methylphenol | 20.12 (1.56) | 19.13 (1.56) | 19.09 (1.77) | 0.262 | 0.153 | 0.997 | 0.157 |
| - 33:63 4-ethylphenol | 13.87 (0.05) | 13.99 (0.39) | 14.17 (0.82) | 0.841 | 0.228 | 0.533 | 0.219 |
| - 38:39 1H-indole^&^ | 19.01 (1.37) | 18.36 (1.71) | 18.35 (1.54) | 0.416 | 0.313 | 1.000 | 0.312 |
| - 41:12 3-methyl-1h-indole^&^ | 15.22 (1.81) | 14.89 (1.77) | 14.92 (1.52) | 0.819 | 0.798 | 0.999 | 0.787 |
| **Alcohols;** **primary**^θ^**; secondary**^ρ^ | | | | | | | |
| - 17:26 Pentan-1-ol^θ^ | 13.30 (2.06) | 12.48 (1.29) | 12.49 (1.39) | 0.243 | 0.167 | 1.000 | 0.161 |
| - 21:21 Hexan-1-ol^ρ^ | 12.24 (0.88) | 12.67 (1.63) | 12.57 (1.40) | 0.607 | 0.683 | 0.960 | 0.606 |
| - 13:28 Butan-1-ol^θ^ | 13.69 (1.76) | 13.86 (1.67) | 13.82 (1.67) | 0.949 | 0.960 | 0.996 | 0.949 |
| - 25:10 Oct-1-en-3-ol^ρ^ | 12.57 (0.45) | 12.96 (0.76) | 12.93 (1.00) | 0.059 | 0.066 | 0.913 | 0.044 |
| - 6:65 Ethanol^θ^ | 15.74 (0.61) | 15.68 (052) | 15.88 (0.77) | 0.967 | 0.737 | 0.524 | 0.507 |
| - 9:54 Propan-1-ol^θ^ | 15.15 (1.91) | 14.85 (1.77) | 15.52 (1.70) | 0.862 | 0.752 | 0.350 | 0.367 |
| **Organosulfur compounds** | | | | | | | |
| - 15:92 (Methyldisulfanyl) methane | 14.41 (1.88) | 14.31 (1.95) | 13.77 (1.84) | 0.988 | 0.473 | 0.543 | 0.390 |
| - 25:22 (Methyltrisulfanyl) methane | 14.13 (1.71) | 14.29 (2.00) | 13.69 (1.77) | 0.961 | 0.690 | 0.459 | 0.441 |
| **Monoterpenes; Terpenoids**^®^ | | | | | | | |
| - 22:55 Alpha-pinene^3^ | 13.23 (1.40) | 12.67 (0.82) | 12.94 (1.04) | 0.253 | 0.615 | 0.620 | 0.285 |
| - 26:10 D-Limonene^4^ | 15.91 (1.04) | 14.56 (1.66) | 14.52 (1.52) | **0.018** | **0.004** | 0.995 | **0.004** |
| - 26:91 Gamma-terpinene^5^ | 14.12 (2.75) | 12.46 (0.94) | 12.63 (1.51) | **0.014** | **0.011** | 0.932 | **0.007** |
| - 28:98 Linalool^6^ | 12.11 (0.96) | 12.04 (1.09) | 12.16 (0.99) | 0.977 | 0.985 | 0.906 | 0.913 |
| - 30:81 Cyclohexa-1,4-diene^®^ | 18.99 (1.20) | 18.92 (1.18) | 18.68 (0.99) | 0.980 | 0.583 | 0.693 | 0.531 |
| **Ester** | | | | | | | |
| - 14:28 Propyl acetate | 13.07 (1.52) | 12.45 (0.16) | 13.26 (1.37) | 0.283 | 0.843 | 0.044 | 0.056 |
| - 24:69 Butyl butanoate | 13.63 (1.54) | 13.25 (1.36) | 13.71 (1.51) | 0.712 | 0.983 | 0.492 | 0.516 |
| - 17:93 Propyl propanoate | 12.41 (1.56) | 12.03 (0.62) | 12.53 (1.31) | 0.626 | 0.938 | 0.306 | 0.337 |
| - 17:55 Ethyl butanoate | 13.25 (1.53) | 13.19 (1.25) | 13.60 (1.56) | 0.992 | 0.694 | 0.571 | 0.523 |
| **Nitrogenous compounds** | | | | | | | |
| - 23:86 Oxime-, methoxy-phenyl-^7^_ | 15.92 (1.17) | 15.57 (0.66) | 15.65 (0.93) | 0.491 | 0.576 | 0.945 | 0.488 |
| **Sesquiterpene; tricyclic**^π^ **or primary**^Ω^ | | | | | | | |
| - 37:56 Alpha-Cubebene^8π^ | 12.79 (1.75) | 12.30 (1.26) | 11.90 (0.68) | 0.404 | **0.023** | 0.407 | **0.028** |
| - 39:51 β-Caryophyllene^9Ω^ | 14.75 (1.62) | 13.55 (1.60) | 13.37 (1.40) | 0.047 | **0.006** | 0.893 | **0.007** |
| **Unclassified** | | | | | | | |
| - 24:31 (1S)-6,6-dimethyl-4-methylidenebicyclo [3.1.1] heptane | 14.80 (1.80) | 12.96 (0.96) | 13.38 (1.49) | **0.001** | **0.003** | 0.539 | **0.001** |

**Notes:**

1. HSD: Tukey's honestly significant difference test
2. One-way ANOVA
3. 2,6,6-trimethyl-bicyclo 3.1.1 hept-2-ene
4. (4R)-1-methyl-4-prop-1-en-2-ylcyclohexene
5. 1-methyl-4-propan-2-ylcyclohexa-1,4-diene
6. 3,7-dimethylocta-1,6-dien-3-ol
7. Methyl-ZN-hydroxy-benzene-carboximidate
8. (1R,5S,6R,7S,10R)-4,10-dimethyl-7-propan-2-yltricyclo [4.4.0.01,5] dec-3-ene_(alpha-Cubebene)
9. (1R,4E,9S)-4,11,11-trimethyl-8-methylidenebicyclo [7.2.0] undec-4-ene_(Caryophyllene)

Statistically-significant differences are shown in bold type

**STable 11: VOCS abundance at baseline and follow-up in Crohn’s disease**

| **Retention time and faecal volatile organic compound** | **Baseline vs follow-up (n=53 pairs)** | | | **Baseline vs remission in follow-up (n=24 pairs)** | | | **Baseline vs active disease in follow-up (n=29 pairs)** | | |
| --- | --- | --- | --- | --- | --- | --- | --- | --- | --- |
|  | **t stat** | **Higher in** | **P value** | **t stat** | **Higher in** | **P value** | **t stat** | **Higher in** | **P-Value^1^** |
| **Ketones or diketones*** | | | | | | | | | |
| - 10:50 Butan-2-one | -0.41 | FUP | 0.69 | -0.42 | FUP | 0.68 | -0.21 | FUP | 0.83 |
| - 10:13 Butane-2,3-dione* | 0.16 | BL | 0.87 | 0.76 | BL | 0.46 | -0.30 | FUP | 0.76 |
| - 13:73 Pentan-2-one | -0.28 | FUP | 0.78 | -0.41 | FUP | 0.69 | 0.01 | BL | 1.00 |
| - 14:06 Pentane-2,3-dione | 0.93 | BL | 0.36 | 0.47 | BL | 0.64 | 0.80 | BL | 0.43 |
| - 17:98 Hexan-2-one | -0.72 | FUP | 0.47 | -0.37 | FUP | 0.72 | -0.63 | FUP | 0.53 |
| - 21:86 Heptan-2-one* | -0.23 | FUP | 0.82 | 0.49 | BL | 0.63 | -0.66 | FUP | 0.52 |
| - 25:56 Octan-2-one | 0.67 | BL | 0.51 | 0.77 | BL | 0.45 | 0.13 | BL | 0.89 |
| - 29:00 Nonan-2-one | -0.66 | FUP | 0.51 | -0.99 | FUP | 0.33 | -0.05 | FUP | 0.96 |
| - 7:37 Propan-2-one (acetone) | -0.94 | FUP | 0.35 | -1.69 | FUP | 0.10 | 0.15 | BL | 0.88 |
| - 16:84 2-Methylpent-1-en-3-one | -0.66 | FUP | 0.51 | 0.18 | BL | 0.86 | -1.07 | FUP | 0.29 |
| - 13:08 3-Methylbut-3-en-2-one | -0.80 | FUP | 0.43 | 0.19 | BL | 0.85 | -1.15 | FUP | 0.26 |
| - 24:71 5-Methylheptan-2-one | -1.27 | FUP | 0.21 | -0.89 | FUP | 0.38 | -0.91 | FUP | 0.37 |
| - 25:46 6-methylhept-5-en-2-one* | 0.68 | BL | 0.50 | -0.35 | FUP | 0.73 | 1.33 | BL | 0.20 |
| **Fatty Acids – short chain; saturated** | | | | | | | | | |
| - 12:43 Acetic acid | -0.90 | FUP | 0.37 | -0.77 | FUP | 0.45 | -0.46 | FUP | 0.65 |
| - 19:45 Butanoic acid | 0.67 | BL | 0.51 | -0.87 | FUP | 0.39 | 1.95 | BL | 0.06 |
| - 16:03 Propanoic acid | 0.69 | BL | 0.49 | -0.57 | FUP | 0.57 | 1.42 | BL | 0.17 |
| **Fatty Acids – branched-chain; saturated**^$^ | | | | | | | | | |
| - 18:15 2-Methylpropanoic acid | -0.69 | FUP | 0.49 | -1.93 | FUP | 0.07 | 0.55 | BL | 0.59 |
| - 21:38 3-Methylbutanoic acid^$^ | -0.03 | FUP | 0.97 | -1.77 | FUP | 0.09 | 1.46 | BL | 0.16 |
| - 21:73 2-Methylbutanoic acid | -0.24 | FUP | 0.81 | -1.71 | FUP | 0.10 | 1.10 | BL | 0.28 |
| - 25:25 4-Methylpentanoic acid | 1.18 | BL | 0.24 | 0.13 | BL | 0.90 | 1.38 | BL | 0.18 |
| **Fatty Acids - medium-chain; saturated** | | | | | | | | | |
| - 22:94 Pentanoic acid | 0.50 | BL | 0.62 | -1.05 | FUP | 0.31 | 1.66 | BL | 0.11 |
| - 26:25 Hexanoic acid | 0.16 | BL | 0.88 | -1.36 | FUP | 0.19 | 1.80 | BL | 0.08 |
| - 29:20 Hepatanoic acid | 0.37 | BL | 0.71 | -0.30 | FUP | 0.77 | 0.90 | BL | 0.38 |
| **Monocarboxylic acid** | | | | | | | | | |
| - 31:48 Cyclohexanecarboxylic acid | 0.64 | BL | 0.53 | -0.24 | FUP | 0.81 | 1.04 | BL | 0.31 |
| **Aldehydes; methylate aldehydes**^α^**; alpha hydrogen**^β^**; aromatic aldehyde**^γ^**γ** | | | | | | | | | |
| - 12:47 3-methylbutanal^α^ | -0.04 | FUP | 0.97 | 1.06 | BL | 0.30 | -0.77 | FUP | 0.45 |
| - 12:72 2-methylbutanal^α^ | -0.59 | FUP | 0.56 | 0.49 | BL | 0.63 | -1.34 | FUP | 0.19 |
| - 18:21 Hexanal | 0.85 | BL | 0.40 | 2.27 | BL | **0.03** | -0.89 | FUP | 0.38 |
| - 22:15 Heptanal | 0.86 | BL | 0.40 | 1.57 | BL | 0.13 | 0.01 | BL | 0.99 |
| - 23:39 3-methylsulfanylpropanal^β^ | -0.06 | FUP | 0.96 | 0.07 | BL | 0.95 | -0.15 | FUP | 0.89 |
| - 25:38 Benzaldehyde^γ^ | 0.55 | BL | 0.59 | 0.46 | BL | 0.65 | 0.34 | BL | 0.74 |
| - 25:88 Octanal | 1.04 | BL | 0.30 | 0.67 | BL | 0.51 | 0.79 | BL | 0.44 |
| - 28:53 2-phenylacetaldehyde | -0.10 | FUP | 0.92 | 0.00 | BL | 1.00 | -0.13 | FUP | 0.89 |
| - 29:39 Nonanal | 1.34 | BL | 0.19 | 0.20 | BL | 0.84 | 1.55 | BL | 0.13 |
| - 9:00 2-methylpropanal^α^ | -0.41 | FUP | 0.68 | 0.12 | BL | 0.90 | -0.69 | FUP | 0.49 |
| **Aromatic compounds; aromatic hydrocarbon**^@^**; aromatic heterocyclic**^&^ | | | | | | | | | |
| - 20:23 Ethylbenzene^@^ | -0.56 | FUP | 0.58 | 0.91 | BL | 0.37 | -1.77 | FUP | 0.09 |
| - 20:53 1,4-xylene | 1.16 | BL | 0.25 | 1.56 | BL | 0.13 | 0.45 | BL | 0.66 |
| - 27:90 Phenol | 2.48 | BL | **0.02** | 1.60 | BL | 0.12 | 1.86 | BL | 0.07 |
| - 30:78 4-methylphenol | -0.70 | FUP | 0.49 | 0.50 | BL | 0.62 | -1.88 | FUP | 0.07 |
| - 33:63 4-ethylphenol | -2.37 | FUP | **0.02** | -1.88 | FUP | 0.07 | -1.48 | FUP | 0.15 |
| - 38:39 1H-indole^&^ | -0.13 | FUP | 0.90 | -0.12 | FUP | 0.91 | -0.07 | FUP | 0.94 |
| - 41:12 3-methyl-1h-indole^&^ | -1.76 | FUP | 0.08 | -2.10 | FUP | 0.05 | -0.37 | FUP | 0.71 |
| **Alcohols;** **primary**^θ^**; secondary**^ρ^ | | | | | | | | | |
| - 17:26 Pentan-1-ol^θ^ | 2.03 | BL | 0.05 | 2.11 | BL | 0.05 | 0.79 | BL | 0.44 |
| - 21:21 Hexan-1-ol^ρ^ | 2.06 | BL | **0.04** | 2.07 | BL | 0.05 | 0.54 | BL | 0.60 |
| - 13:28 Butan-1-ol^θ^ | 0.73 | BL | 0.47 | 0.26 | BL | 0.80 | 0.75 | BL | 0.46 |
| - 25:10 Oct-1-en-3-ol^ρ^ | 0.23 | BL | 0.82 | -0.31 | FUP | 0.76 | 0.62 | BL | 0.54 |
| - 6:65 Ethanol^θ^ | 2.11 | BL | **0.04** | 1.25 | BL | 0.22 | 1.72 | BL | 0.10 |
| - 9:54 Propan-1-ol^θ^ | 4.91 | BL | **<0.001** | 3.43 | BL | **<0.01** | 3.47 | BL | **<0.01** |
| **Organosulfur compounds** | | | | | | | | | |
| - 15:92 (Methyldisulfanyl) methane | -1.29 | FUP | 0.20 | -0.87 | FUP | 0.39 | -0.95 | FUP | 0.35 |
| - 25:22 (Methyltrisulfanyl) methane | -0.73 | FUP | 0.47 | -0.60 | FUP | 0.55 | -0.44 | FUP | 0.66 |
| **Monoterpenes; Terpenoids**^®^ | | | | | | | | | |
| - 22:55 Alpha-pinene^2^ | 1.11 | BL | 0.27 | 2.06 | BL | 0.05 | -0.44 | FUP | 0.66 |
| - 26:10 D-Limonene^3^ | 0.26 | BL | 0.79 | 0.53 | BL | 0.60 | -0.12 | FUP | 0.90 |
| - 26:91 Gamma-terpinene^4^ | 1.03 | BL | 0.31 | 0.78 | BL | 0.44 | 0.67 | BL | 0.51 |
| - 28:98 Linalool^5^ | -0.93 | FUP | 0.36 | -1.15 | FUP | 0.26 | -0.28 | FUP | 0.78 |
| - 30:81 Cyclohexa-1,4-diene^®^ | -1.22 | FUP | 0.23 | -2.02 | FUP | 0.06 | 0.15 | BL | 0.88 |
| **Ester** | | | | | | | | | |
| - 14:28 Propyl acetate | -0.17 | FUP | 0.87 | -1.09 | FUP | 0.29 | 0.91 | BL | 0.37 |
| - 24:69 Butyl butanoate | -0.10 | FUP | 0.92 | -1.16 | FUP | 0.26 | 0.80 | BL | 0.43 |
| - 17:93 Propyl propanoate | -0.04 | FUP | 0.97 | -2.10 | FUP | 0.05 | 1.63 | BL | 0.11 |
| - 17:55 Ethyl butanoate | 0.44 | BL | 0.66 | -1.11 | FUP | 0.28 | 1.40 | BL | 0.17 |
| **Nitrogenous compounds** | | | | | | | | | |
| - 23:86 Oxime-, methoxy-phenyl-^6^_ | -0.29 | FUP | 0.77 | 0.41 | BL | 0.69 | -0.67 | FUP | 0.51 |
| **Sesquiterpene; tricyclic**^π^ **or primary**^Ω^ | | | | | | | | | |
| - 37:56 Alpha-Cubebene^7π^ | 1.51 | BL | 0.14 | 1.41 | BL | 0.17 | 0.80 | BL | 0.43 |
| - 39:51 β-Caryophyllene^8Ω^ | -0.36 | FUP | 0.72 | 0.57 | BL | 0.57 | -1.07 | FUP | 0.29 |
| **Unclassified** | | | | | | | | | |
| - 24:31 (1S)-6,6-dimethyl-4-methylidenebicyclo [3.1.1] heptane | -0.26 | FUP | 0.80 | 0.83 | BL | 0.41 | -1.34 | FUP | 0.19 |

**Notes:**

1. A paired t-test
2. 2,6,6-trimethyl-bicyclo 3.1.1 hept-2-ene
3. (4R)-1-methyl-4-prop-1-en-2-ylcyclohexene
4. 1-methyl-4-propan-2-ylcyclohexa-1,4-diene
5. 3,7-dimethylocta-1,6-dien-3-ol
6. Methyl-ZN-hydroxy-benzene-carboximidate
7. (1R,5S,6R,7S,10R)-4,10-dimethyl-7-propan-2-yltricyclo [4.4.0.01,5] dec-3-ene_(alpha-Cubebene)
8. (1R,4E,9S)-4,11,11-trimethyl-8-methylidenebicyclo [7.2.0] undec-4-ene_(Caryophyllene)

Statistically-significant differences are shown in bold type

**STable 12: Associations between dietary therapy and VOCs abundance in 19 children with CD at FUP^1^**

| **Retention time and Faecal volatile organic compound** | **s0** |
| --- | --- |
| **(Intercept)** | 1.46 |
| **Ketones or diketones*** | |
| - 10:50 Butan-2-one | 0 |
| - 10:13 Butane-2,3-dione* | -0.14 |
| - 13:73 Pentan-2-one | 0 |
| - 14:06 Pentane-2,3-dione | 0 |
| - 17:98 Hexan-2-one | 0 |
| - 21:86 Heptan-2-one* | 0 |
| - 25:56 Octan-2-one | 0 |
| - 29:00 Nonan-2-one | 0 |
| - 7:37 Propan-2-one (acetone) | 0 |
| - 16:84 2-Methylpent-1-en-3-one | 0 |
| - 13:08 3-Methylbut-3-en-2-one | -0.82 |
| - 24:71 5-Methylheptan-2-one | 0 |
| - 25:46 6-methylhept-5-en-2-one* | 0 |
| **Fatty Acids– short-chain; saturated** | |
| - 12:43 Acetic acid | 0 |
| - 19:45 Butanoic acid | 0 |
| - 16:03 Propanoic acid | 0 |
| **Fatty Acids – branched-chain/ saturated**^$^ | |
| - 18:15 2-Methylpropanoic acid | 0 |
| - 21:38 3-Methylbutanoic acid^$^ | 0 |
| - 21:73 2-Methylbutanoic acid | 0 |
| - 25:25 4-Methylpentanoic acid | 0 |
| **Fatty Acids – medium-chain; saturated** | |
| - 22:94 Pentanoic acid | -0.37 |
| - 26:25 Hexanoic acid | 0 |
| - 29:20 Hepatanoic acid | 0 |
| **Mono carboxylic acid** | |
| - 31:48 Cyclohexanecarboxylic acid | 0 |
| **Aldehydes; methylate aldehydes**^α^**; alpha hydrogen**^β^**; aromatic aldehyde**^γ^ | |
| - 12:47 3-methylbutanal^α^ | 0 |
| - 12:72 2-methylbutanal^α^ | 0.20 |
| - 18:21 Hexanal | 0 |
| - 22:15 Heptanal | 0 |
| - 23:39 3-methylsulfanylpropanal^β^ | 0 |
| 25:38 Benzaldehyde^γ^ | 0 |
| - 25:88 Octanal | 0 |
| - 28:53 2-phenylacetaldehyde | 0 |
| - 29:39 Nonanal | 1.27 |
| - 9:00 2-methylpropanal^α^ | 0 |
| **Aromatic compounds; aromatic hydrocarbon**^@^**; aromatic heterocyclic**^&^ | |
| - 20:23 Ethylbenzene^@^ | 0 |
| - 20:53 1,4-xylene | 0 |
| - 27:90 Phenol | 0 |
| - 30:78 4-methylphenol | 0 |
| - 33:63 4-ethylphenol | 0 |
| - 38:39 1H-indole^&^ | 0 |
| - 41:12 3-methyl-1h-indole^&^ | 0 |
| **Alcohols;** **primary**^θ^**; secondary**^ρ^ | |
| - 17:26 Pentan-1-ol^θ^ | 0 |
| - 21:21 Hexan-1-ol^ρ^ | -0.028 |
| - 13:28 Butan-1-ol^θ^ | 0 |
| 25:10 Oct-1-en-3-ol^ρ^ | 0 |
| - 6:65 Ethanol^θ^ | 0 |
| - 9:54 Propan-1-ol^θ^ | 0 |
| **Organosulfur compounds** | |
| - 15:92 (Methyldisulfanyl) methane | 0 |
| - 25:22 (Methyltrisulfanyl) methane | 0 |
| **Monoterpenes or Terpenoids**^®^ | |
| - 22:55 Alpha-pinene^2^ | 0 |
| - 26:10 D-Limonene^3^ | 0 |
| - 26:91 Gamma-terpinene^4^ | 0 |
| - 28:98 Linalool^5^ | 0 |
| 30:81 Cyclohexa-1,4-diene^®^ | 0 |
| **Ester** | |
| - 14:28 Propyl acetate | 0 |
| - 24:69 Butyl butanoate | 0 |
| - 17:93 Propyl propanoate | 0 |
| - 17:55 Ethyl butanoate | 0 |
| **Nitrogenous compounds** | |
| - 23:86 Oxime-, methoxy-phenyl-^6^ | 0 |
| **Sesquiterpene; tricyclic**^π^ **or primary**^Ω^ |  |
| - 37:56 Alpha-Cubebene^7π^ | 0 |
| - 39:51 β-Caryophyllene^8Ω^ | -0.034 |
| **Unclassified** | |
| - 24:3 1(1S)-6,6-dimethyl-4-methylidenebicyclo [3.1.1] heptane | 0 |

**Notes:**

1. Performed by LASSO regression; l1-panelty constraint forces non-significant beta coefficients to go to zero leaving only those that are changing with respect to the parameter of interest
2. 2,6,6-trimethyl-bicyclo 3.1.1 hept-2-ene
3. (4R)-1-methyl-4-prop-1-en-2-ylcyclohexene
4. 1-methyl-4-propan-2-ylcyclohexa-1,4-diene
5. 3,7-dimethylocta-1,6-dien-3-ol
6. Methyl-ZN-hydroxy-benzene-carboximidate
7. (1R,5S,6R,7S,10R)-4,10-dimethyl-7-propan-2-yltricyclo [4.4.0.01,5] dec-3 ene_ (alpha-Cubebene)
8. (1R,4E,9S)-4,11,11-trimethyl-8-methylidenebicyclo [7.2.0] undec-4-ene_(Caryophyllene)

**STable 13: Difference in faecal volatile organic compounds abundance in the ulcerative colitis/matched controls pairs**

| **Retention time and Faecal volatile organic compound** | **Median (IQR) difference^1^** | **P-Value^2^** |
| --- | --- | --- |
| **Ketones or diketones*** | | |
| - 10:50 Butan-2-one | -1.41 (-2.49- 0.72) | **0.027** |
| - 10:13 Butane-2,3-dione* | -0.54 (-1.79- 0.27) | **0.010** |
| - 13:73 Pentan-2-one | -0.07 (-1.34- 1.33) | 0.898 |
| - 14:06 Pentane-2,3-dione | -1.11 (-2.37- 0.67) | **0.041** |
| - 17:98 Hexan-2-one | -0.72 (-2.33- 0.72) | **0.031** |
| - 21:86 Heptan-2-one* | -1.45 (-2.89- 0.79) | **0.004** |
| - 25:56 Octan-2-one | -0.27 (-1.04- 0.00) | **0.006** |
| - 29:00 Nonan-2-one | 0.00 (-1.99- 0.23) | **0.028** |
| - 7:37 Propan-2-one (acetone) | 0.00 (-1.47- 1.63) | 0.888 |
| - 16:84 2-Methylpent-1-en-3-one | -0.74 (-1.04- 0.87) | 0.365 |
| - 13:08 3-Methylbut-3-en-2-one | 0.00 (-0.52- 0.52) | 0.944 |
| - 24:71 5-Methylheptan-2-one | -0.29 (-2.06- 0.17) | **0.008** |
| - 25:46 6-methylhept-5-en-2-one* | -0.69 (-3.11- 0.26) | **0.007** |
| **Fatty Acids– short-chain; saturated** | | |
| - 12:43 Acetic acid | 0.42 (-2.45- 2.98) | 0.544 |
| - 19:45 Butanoic acid | -0.24 (-3.01- 0.85) | 0.300 |
| - 16:03 Propanoic acid | -0.28 (-3.28- 1.19) | 0.194 |
| **Fatty Acids – branched-chain/ saturated**^$^ | | |
| - 18:15 2-Methylpropanoic acid | 0.02 (-3.29- 0.79) | 0.429 |
| - 21:38 3-Methylbutanoic acid^$^ | -0.09 (-3.29- 0.83) | 0.180 |
| - 21:73 2-Methylbutanoic acid | -0.64 (-3.52- 0.65) | **0.020** |
| - 25:25 4-Methylpentanoic acid | 0.41 (-2.31- 2.40) | 0.540 |
| **Fatty Acids – medium-chain; saturated** |  |  |
| - 22:94 Pentanoic acid | -1.08 (-3.43- 0.94) | **0.010** |
| - 26:25 Hexanoic acid | -0.65 (-3.27- 1.11) | 0.076 |
| - 29:20 Hepatanoic acid | 0.00 (-0.64- 0.64) | 0.447 |
| **Mono carboxylic acid** |  |  |
| - 31:48 Cyclohexanecarboxylic acid | -0.20 (-1.93- 0.20) | 0.106 |
| **Aldehydes; methylate aldehydes**^α^**; alpha hydrogen**^β^**; aromatic aldehyde**^γ^ | | |
| - 12:47 3-methylbutanal^α^ | 0.29 (-1.15- 1.94) | 0.342 |
| - 12:72 2-methylbutanal^α^ | -0.33 (-1.73- 1.14) | 0.220 |
| - 18:21 Hexanal | 0.45 (-0.99- 1.92) | 0.577 |
| - 22:15 Heptanal | -0.41 (-1.29- 0.70) | 0.120 |
| - 23:39 3-methylsulfanylpropanal^β^ | -1.05 (-2.57- 0.10) | **0.004** |
| 25:38 Benzaldehyde^γ^ | -0.66 (-1.89- 1.02) | 0.198 |
| - 25:88 Octanal | -0.36 (-2.12- 0.42) | **0.038** |
| - 28:53 2-phenylacetaldehyde | -0.46 (-2.27- 0.89) | 0.141 |
| - 29:39 Nonanal | -0.05 (-2.05- 0.01) | **0.027** |
| - 9:00 2-methylpropanal^α^ | 0.07 (-1.39- 1.03) | 0.806 |
| **Aromatic compounds; aromatic hydrocarbon**^@^**; aromatic heterocyclic**^&^ | | |
| - 20:23 Ethylbenzene^@^ | -0.29 (-1.52- 0.00) | **0.002** |
| - 20:53 1,4-xylene | -0.34 (-0.34- 0.00) | 0.192 |
| - 27:90 Phenol | -0.24 (-1.67- 2.84) | 0.421 |
| - 30:78 4-methylphenol | -2.04 (-4.35- 0.36) | **<0.001** |
| - 33:63 4-ethylphenol | 0.00 (-0.82- 0.09) | 0.059 |
| - 38:39 1H-indole^&^ | -0.42 (-1.62- 0.65) | 0.137 |
| - 41:12 3-methyl-1h-indole^&^ | -0.72 (-2.51- 0.77) | 0.124 |
| **Alcohols;** **primary**^θ^**; secondary**^ρ^ | | |
| - 17:26 Pentan-1-ol^θ^ | -0.17 (-1.99- 0.00) | **0.007** |
| - 21:21 Hexan-1-ol^ρ^ | 0.00 (-0.53- 0.45) | 0.566 |
| - 13:28 Butan-1-ol^θ^ | -0.24 (-1.99- 0.06) | 0.101 |
| 25:10 Oct-1-en-3-ol^ρ^ | 0.44 (0.00- 0.88) | **0.012** |
| - 6:65 Ethanol^θ^ | 0.00 (-0.54- 0.73) | 0.580 |
| - 9:54 Propan-1-ol^θ^ | 0.29 (-0.81- 1.84) | 0.054 |
| **Organosulfur compounds** | | |
| - 15:92 (Methyldisulfanyl) methane | -0.70 (-3.74- 0.39) | **0.010** |
| - 25:22 (Methyltrisulfanyl) methane | -0.07 (-3.76- 1.01) | 0.062 |
| **Monoterpenes or Terpenoids**^®^ | | |
| - 22:55 Alpha-pinene^3^ | -0.05 (-1.65- 0.13) | 0.293 |
| - 26:10 D-Limonene^4^ | -0.99 (-3.48- 0.13) | **0.003** |
| - 26:91 Gamma-terpinene^5^ | -0.07 (-1.38- 0.00) | **0.003** |
| - 28:98 Linalool^6^ | 0.00 (-0.24- 0.00) | 0.090 |
| 30:81 Cyclohexa-1,4-diene^®^ | 0.00 (-0.30- 0.30) | 0.902 |
| **Ester** | | |
| - 14:28 Propyl acetate | 0.00 (-0.31- 0.31) | 0.919 |
| - 24:69 Butyl butanoate | 0.00 (-0.42- 0.17) | 0.665 |
| - 17:93 Propyl propanoate | 0.00 (-0.22- 0.22) | 0.753 |
| - 17:55 Ethyl butanoate | 0.00 (-0.25- 1.22) | 0.293 |
| **Nitrogenous compounds** | | |
| - 23:86 Oxime-, methoxy-phenyl-^7^ | 0.00 (-0.43- 0.61) | 0.829 |
| **Sesquiterpene; tricyclic**^π^ **or primary**^Ω^ | | |
| - 37:56 Alpha-Cubebene^8π^ | 0.00 (-1.29- 0.09) | 0.112 |
| - 39:51 β-Caryophyllene^9Ω^ | -1.39 (-2.43- 0.64) | **0.007** |
| **Unclassified** | | |
| - 24:31 (1S)-6,6-dimethyl-4-methylidenebicyclo [3.1.1] heptane | -0.45 (-2.79- 0.74) | **0.046** |

**Notes:**

1. A negative value occurs when VOCs abundance is lower in cases than controls and *vice versa*
2. Wilcoxon Signed Rank Test
3. 2,6,6-trimethyl-bicyclo 3.1.1 hept-2-ene
4. (4R)-1-methyl-4-prop-1-en-2-ylcyclohexene
5. 1-methyl-4-propan-2-ylcyclohexa-1,4-diene
6. 3,7-dimethylocta-1,6-dien-3-ol
7. Methyl-ZN-hydroxy-benzene-carboximidate
8. (1R,5S,6R,7S,10R)-4,10-dimethyl-7-propan-2-yltricyclo [4.4.0.01,5] dec-3 ene_ (alpha-Cubebene
9. (1R,4E,9S)-4,11,11-trimethyl-8-methylidenebicyclo [7.2.0] undec-4-ene_(Caryophyllene)

Statistically-significant differences are shown in bold type

**STable 14: Faecal volatile organic compounds according to disease severity in colitis**

| **Retention time and faecal volatile organic compound** | **Mild + Remission**  **Mean (SD)**  **N= 15** | **Moderate**  **Mean (SD)**  **N= 28** | **Severe**  **Mean (SD)**  **N= 11** | **HSD^1^ Moderate- Mild** | **HSD Severe- Mild** | **HSD Severe-Moderate** | **P-Value^2^** |
| --- | --- | --- | --- | --- | --- | --- | --- |
| **Ketones or diketones*** | | | | | | | |
| - 10:50 Butan-2-one | 14.47 (1.64) | 13.68 (1.92) | 13.23 (1.07) | 0.338 | 0.179 | 0.738 | 0.182 |
| - 10:13 Butane-2,3-dione* | 13.65 (1.30) | 14.51 (1.10) | 13.31 (1.19) | 0.070 | 0.759 | 0.015 | **0.008** |
| - 13:73 Pentan-2-one | 15.11 (1.29) | 14.44 (1.43) | 13.76 (1.28) | 0.296 | 0.045 | 0.342 | 0.056 |
| - 14:06 Pentane-2,3-dione | 14.57 (1.26) | 15.01 (1.88) | 13.69 (1.54) | 0.710 | 0.397 | 0.078 | 0.095 |
| - 17:98 Hexan-2-one | 13.71 (1.92) | 13.22 (1.33) | 12.88 (0.94) | 0.555 | 0.333 | 0.782 | 0.350 |
| - 21:86 Heptan-2-one* | 14.76 (1.91) | 13.63 (1.57) | 12.91 (0.78) | 0.075 | **0.012** | 0.389 | **0.014** |
| - 25:56 Octan-2-one | 13.56 (1.33) | 13.37 (1.41) | 12.82 (0.14) | 0.885 | 0.311 | 0.433 | 0.320 |
| - 29:00 Nonan-2-one | 13.03 (1.12) | 12.44 (0.71) | 12.58 (0.78) | 0.091 | 0.395 | 0.881 | 0.109 |
| - 7:37 Propan-2-one (acetone) | 14.02 (1.24) | 14.45 (1.22) | 16.20 (3.45) | 0.757 | **0.016** | 0.031 | **0.014** |
| - 16:84 2-Methylpent-1-en-3-one | 12.92 (1.85) | 11.96 (1.12) | 11.42 (0.39) | 0.061 | **0.013** | 0.449 | **0.013** |
| - 13:08 3-Methylbut-3-en-2-one | 14.19 (0.73) | 13.91 (0.48) | 13.86 (0.27) | 0.246 | 0.271 | 0.955 | 0.204 |
| - 24:71 5-Methylheptan-2-one | 13.06 (1.20) | 13.06 (1.32) | 12.84 (1.24) | 1.000 | 0.903 | 0.884 | 0.882 |
| - 25:46 6-methylhept-5-en-2-one* | 15.23 (1.23) | 13.97 (1.56) | 12.85 (0.46) | **0.014** | **<0.001** | 0.054 | **<0.001** |
| **Fatty Acids – short chain; saturated** | | | | | | | |
| - 12:43 Acetic acid | 16.83 (2.06) | 17.09 (2.03) | 15.15 (2.28) | 0.922 | 0.125 | **0.031** | **0.037** |
| - 19:45 Butanoic acid | 19.71 (1.09) | 19.32 (1.55) | 16.21 (3.29) | 0.811 | 0.000 | **<0.001** | **<0.001** |
| - 16:03 Propanoic acid | 18.03 (1.20) | 17.20 (1.49) | 14.94 (2.99) | 0.346 | **<0.001** | **0.003** | **<0.001** |
| **Fatty Acids – branched-chain; saturated**^$^ | | | | | | | |
| - 18:15 2-Methylpropanoic acid | 17.64 (1.50) | 16.70 (1.91) | 14.66 (2.63) | 0.317 | **0.001** | **0.015** | **0.002** |
| - 21:38 3-Methylbutanoic acid**^$^** | 18.74 (1.56) | 18.17 (1.92) | 15.31 (3.08) | 0.693 | **0.001** | **0.001** | **<0.001** |
| - 21:73 2-Methylbutanoic acid | 18.30 (1.71) | 17.63 (1.82) | 15.24 (2.81) | 0.573 | **0.001** | **0.005** | **0.001** |
| - 25:25 4-Methylpentanoic acid | 15.60 (2.10) | 15.52 (2.27) | 13.87 (2.09) | 0.993 | 0.135 | 0.096 | 0.088 |
| **Fatty Acids – medium-chain; saturated** | | | | | | | |
| - 22:94 Pentanoic acid | 18.80 (1.35) | 18.02 (1.79) | 15.48 (2.95) | 0.457 | **<0.001** | **0.002** | **<0.001** |
| - 26:25 Hexanoic acid | 16.55 (2.61) | 16.33 (2.27) | 14.61 (1.81) | 0.954 | 0.097 | 0.093 | 0.073 |
| - 29:20 Hepatanoic acid | 15.28 (2.07) | 14.83 (0.90) | 14.58 (0.52) | 0.520 | 0.361 | 0.843 | 0.363 |
| **Monocarboxylic acid** | | | | | | | |
| - 31:48 Cyclohexanecarboxylic acid | 13.85 (1.41) | 13.94 (1.64) | 13.29 (0.76) | 0.982 | 0.599 | 0.418 | 0.442 |
| **Aldehydes; methylate aldehydes**^α^**; alpha hydrogen**^β^**; aromatic aldehyde**^γ^ | | | | | | | |
| - 12:47 3-methylbutanal^α^ | 16.03 (0.89) | 15.62 (1.96) | 14.63 (1.97) | 0.758 | 0.127 | 0.255 | 0.138 |
| - 12:72 2-methylbutanal^α^ | 15.13 (1.61) | 14.95 (2.06) | 14.06 (1.92) | 0.954 | 0.361 | 0.404 | 0.343 |
| - 18:21 Hexanal | 13.27(1.53) | 13.17 (1.60) | 13.45 (2.14) | 0.982 | 0.962 | 0.888 | 0.897 |
| - 22:15 Heptanal | 13.24 (0.96) | 12.81 (1.20) | 12.18 (0.91) | 0.455 | 0.051 | 0.242 | 0.064 |
| - 23:39 3-methylsulfanylpropanal^β^ | 13.34 (1.28) | 12.77 (1.29) | 11.81 (0.73) | 0.321 | **0.007** | 0.070 | **0.010** |
| - 25:38 Benzaldehyde^γ^ | 15.66 (1.13) | 14.73 (1.43) | 14.04 (1.67) | 0.117 | **0.017** | 0.358 | **0.020** |
| - 25:88 Octanal | 13.47 (1.14) | 12.92 (1.23) | 11.96 (0.61) | 0.289 | **0.004** | 0.047 | **0.006** |
| - 28:53 2-phenylacetaldehyde | 16.80 (0.92) | 16.10 (1.55) | 14.50 (1.96) | 0.330 | **0.001** | **0.012** | **0.001** |
| - 29:39 Nonanal | 12.63 (0.98) | 12.33 (0.88) | 12.23 (0.62) | 0.543 | 0.486 | 0.939 | 0.456 |
| - 9:00 2-methylpropanal^α^ | 13.46 (0.63) | 13.40 (1.05) | 13.00 (1.08) | 0.978 | 0.467 | 0.479 | 0.436 |
| **Aromatic compounds; aromatic hydrocarbon**^@^**; aromatic heterocyclic**^&^ | | | | | | | |
| - 20:23 Ethylbenzene^@^ | 12.18 (1.07) | 12.04 (1.08) | 11.81 (0.53) | 0.897 | 0.620 | 0.790 | 0.646 |
| - 20:53 1,4-xylene | 12.05 (0.75) | 12.29 (1.17) | 11.85 (0.18) | 0.728 | 0.855 | 0.397 | 0.401 |
| - 27:90 Phenol | 18.00 (1.92) | 16.34 (2.35) | 15.51 (3.22) | 0.103 | **0.039** | 0.608 | **0.036** |
| - 30:78 4-methylphenol | 19.30 (1.81) | 18.12 (2.13) | 16.88 (2.48) | 0.218 | **0.018** | 0.234 | **0.025** |
| - 33:63 4-ethylphenol | 14.03 (0.53) | 14.04 (0.62) | 14.00 (0.44) | 0.999 | 0.986 | 0.975 | 0.977 |
| - 38:39 1H-indole^&^ | 18.40 (1.40) | 17.71 (1.56) | 17.04 (1.96) | 0.388 | 0.098 | 0.469 | 0.116 |
| - 41:12 3-methyl-1h-indole^&^ | 15.70 (1.97) | 14.68 (1.67) | 14.28 (1.42) | 0.167 | 0.107 | 0.789 | 0.092 |
| **Alcohols;** **primary**^θ^**; secondary**^ρ^ | | | | | | | |
| - 17:26 Pentan-1-ol^θ^ | 12.04 (0.87) | 12.06 (0.89) | 12.10 (0.80) | 0.998 | 0.985 | 0.991 | 0.986 |
| - 21:21 Hexan-1-ol^ρ^ | 12.45 (1.18) | 12.20 (0.74) | 12.36 (1.00) | 0.674 | 0.968 | 0.869 | 0.675 |
| - 13:28 Butan-1-ol^θ^ | 13.43 (1.66) | 13.51 (1.73) | 13.01 (1.15) | 0.988 | 0.789 | 0.652 | 0.673 |
| - 25:10 Oct-1-en-3-ol^ρ^ | 12.85 (0.83) | 13.12 (1.28) | 12.95 (0.65) | 0.707 | 0.966 | 0.897 | 0.713 |
| - 6:65 Ethanol^θ^ | 15.41 (0.76) | 16.04 (1.54) | 15.48 (0.60) | 0.272 | 0.990 | 0.411 | 0.215 |
| - 9:54 Propan-1-ol^θ^ | 14.04 (2.03) | 15.20 (1.89) | 14.29 (1.92) | 0.164 | 0.944 | 0.385 | 0.140 |
| **Organosulfur compounds** | | | | | | | |
| - 15:92 (Methyldisulfanyl) methane | 14.29 (2.02) | 14.03 (2.25) | 13.04 (1.21) | 0.914 | 0.281 | 0.359 | 0.276 |
| - 25:22 (Methyltrisulfanyl) methane | 14.17 (2.02) | 13.57 (2.22) | 12.88 (1.43) | 0.641 | 0.266 | 0.605 | 0.298 |
| **Monoterpenes; Terpenoids**^®^ | | | | | | | |
| - 22:55 Alpha-pinene^3^ | 12.62 (0.60) | 12.90 (0.94) | 12.91 (1.36) | 0.655 | 0.744 | 1.000 | 0.650 |
| - 26:10 D-Limonene^4^ | 15.44 (1.30) | 14.31 (2.17) | 13.76 (2.51) | 0.225 | 0.119 | 0.732 | 0.114 |
| - 26:91 Gamma-terpinene^5^ | 12.40 (1.24) | 12.37 (1.05) | 12.27 (1.10) | 0.995 | 0.957 | 0.970 | 0.959 |
| - 28:98 Linalool^6^ | 11.91 (0.81) | 11.87 (0.68) | 11.84 (0.64) | 0.990 | 0.971 | 0.990 | 0.973 |
| - 30:81 Cyclohexa-1,4-diene^®^ | 18.83 (1.18) | 18.38 (0.75) | 18.33 (0.58) | 0.240 | 0.311 | 0.983 | 0.216 |
| **Ester** | | | | | | | |
| - 14:28 Propyl acetate | 12.79 (1.00) | 13.38 (1.62) | 12.72 (0.81) | 0.374 | 0.992 | 0.361 | 0.248 |
| - 24:69 Butyl butanoate | 13.15 (1.25) | 13.67 (2.00) | 12.91 (1.03) | 0.614 | 0.929 | 0.409 | 0.375 |
| - 17:93 Propyl propanoate | 12.28 (1.07) | 12.89 (1.56) | 12.34 (0.98) | 0.354 | 0.995 | 0.480 | 0.289 |
| - 17:55 Ethyl butanoate | 13.07 (1.10) | 14.13 (2.11) | 12.95 (0.81) | 0.139 | 0.983 | 0.129 | 0.062 |
| **Nitrogenous compounds** | | | | | | | |
| - 23:86 Oxime-, methoxy-phenyl-^7^_ | 15.86 (0.86) | 15.77 (0.62) | 15.52 (0.52) | 0.916 | 0.419 | 0.533 | 0.427 |
| **Sesquiterpene; tricyclic**^π^ **or primary**^Ω^ | | | | | | | |
| - 37:56 Alpha-Cubebene^8π^ | 12.48 (1.46) | 11.86 (0.63) | 11.60 (0.05) | 0.084 | **0.042** | 0.690 | **0.035** |
| - 39:51 β-Caryophyllene^9Ω^ | 14.22 (1.45) | 13.15 (1.36) | 12.60 (0.97) | **0.040** | **0.010** | 0.474 | **0.009** |
| **Unclassified** | | | | | | | |
| - 24:31 (1S)-6,6-dimethyl-4-methylidenebicyclo [3.1.1] heptane | 13.73 (1.25) | 13.22 (1.26) | 13.12 (1.60) | 0.459 | 0.493 | 0.979 | 0.417 |

**Notes:**

1. HSD: Tukey's honestly significant difference test
2. One-way ANOVA
3. 2,6,6-trimethyl-bicyclo 3.1.1 hept-2-ene
4. (4R)-1-methyl-4-prop-1-en-2-ylcyclohexene
5. 1-methyl-4-propan-2-ylcyclohexa-1,4-diene
6. 3,7-dimethylocta-1,6-dien-3-ol
7. Methyl-ZN-hydroxy-benzene-carboximidate
8. (1R,5S,6R,7S,10R)-4,10-dimethyl-7-propan-2-yltricyclo [4.4.0.01,5] dec-3-ene_(alpha-Cubebene)
9. (1R,4E,9S)-4,11,11-trimethyl-8-methylidenebicyclo [7.2.0] undec-4-ene_(Caryophyllene)

Statistically-significant differences are shown in bold type

**STable 15: Faecal volatile organic compounds according to disease distribution in colitis**

| **Retention time and faecal volatile organic compound** | **Distal inflammation**  **(E1+E2)**  **N=15**  **Mean (SD)** | **Extensive colitis**  **(E3+E4)**  **N=39**  **Mean (SD)** | **P-Value^1^** |
| --- | --- | --- | --- |
| **Ketones or diketones*** | | | |
| - 10:50 Butan-2-one | 14.54 (2.14) | 13.51 (1.49) | 0.101 |
| - 10:13 Butane-2,3-dione* | 14.21 (1.58) | 13.98 (1.13) | 0.604 |
| - 13:73 Pentan-2-one | 14.47 (1.51) | 14.48 (1.40) | 0.988 |
| - 14: 06 Pentane-2,3-dione | 15.42 (2.02) | 14.32 (1.51) | 0.069 |
| - 17:98 Hexan-2-one | 13.73 (1.46) | 13.10 (1.42) | 0.165 |
| - 21:86 Heptan-2-one* | 14.46 (1.87) | 13.51 (1.51) | 0.094 |
| - 25:56 Octan-2-one | 13.84 (1.77) | 13.10 (0.93) | 0.141 |
| - 29:00 Nonan-2-one | 12.65 (0.75) | 12.61 (0.92) | 0.888 |
| - 7:37 Propan-2-one (acetone) | 14.77 (1.40) | 14.67 (2.22) | 0.848 |
| - 16:84 2-Methylpent-1-en-3-one | 12.55 (1.83) | 11.93 (1.09) | 0.231 |
| - 13:08 3-Methylbut-3-en-2-one | 14.39 (0.80) | 13.82 (0.26) | **0.016** |
| - 24:71 5-Methylheptan-2-one | 13.32 (1.34) | 12.90 (1.21) | 0.296 |
| - 25:46 6-methylhept-5-en-2-one* | 15.03 (1.63) | 13.69 (1.34) | **0.010** |
| **Fatty Acids – short chain; saturated** | | | |
| - 12:43 Acetic acid | 17.07 (1.61) | 16.46 (2.37) | 0.281 |
| - 19:45 Butanoic acid | 19.04 (1.86) | 18.69 (2.48) | 0.573 |
| - 16:03 Propanoic acid | 17.15 (1.65) | 16.88 (2.26) | 0.641 |
| **Fatty Acids – branched-chain; saturated**^$^ | | | |
| - 18:15 2-Methylpropanoic acid | 17.25 (1.65) | 16.25 (2.34) | 0.088 |
| - 21:38 3-Methylbutanoic acid^$^ | 18.15 (1.92) | 17.58 (2.61) | 0.386 |
| - 21:73 2-Methylbutanoic acid | 17.83 (1.94) | 17.12 (2.38) | 0.265 |
| - 25:25 4-Methylpentanoic acid | 15.41 (2.37) | 15.12 (2.23) | 0.693 |
| **Fatty Acids – Medium-chain/ saturated** | | | |
| - 22:94 Pentanoic acid | 18.13 (1.99) | 17.54 (2.38) | 0.366 |
| - 26:25 Hexanoic acid | 16.77 (2.46) | 15.76 (2.29) | 0.181 |
| - 29:20 Hepatanoic acid | 15.04 (1.53) | 14.84 (1.16) | 0.653 |
| **Monocarboxylic acid** | | | |
| - 31:48 Cyclohexanecarboxylic acid | 13.27 (0.83) | 13.98 (1.58) | **0.036** |
| **Aldehydes; methylate aldehydes**^α^**; alpha hydrogen**^β^**; aromatic aldehyde**^γ^ | | | |
| - 12:47 3-methylbutanal^α^ | 16.10 (1.19) | 15.31 (1.94) | 0.074 |
| - 12:72 2-methylbutanal^α^ | 15.14 (1.75) | 14.69 (2.01) | 0.428 |
| - 18:21 Hexanal | 12.78 (1.46) | 13.44 (1.73) | 0.170 |
| - 22:15 Heptanal | 12.95 (1.26) | 12.73 (1.08) | 0.546 |
| - 23:39 3-methylsulfanylpropanal^β^ | 13.02 (1.52) | 12.61 (1.19) | 0.351 |
| - 25:38 Benzaldehyde^γ^ | 15.34 (1.26) | 14.64 (154) | 0.096 |
| - 25:88 Octanal | 13.04 (1.28) | 12.80 (1.18) | 0.537 |
| - 28:53 2-phenylacetaldehyde | 16.57 (1.41) | 15.72 (1.74) | 0.072 |
| - 29:39 Nonanal | 12.63 (1.04) | 12.30 (0.77) | 0.278 |
| - 9:00 2-methylpropanal^α^ | 13.65 (1.23) | 13.21 (0.82) | 0.24 |
| **Aromatic compounds; aromatic hydrocarbon**^@^**; aromatic heterocyclic**^&^ | | | |
| - 20:23 Ethylbenzene^@^ | 12.22 (1.23) | 11.95 (0.83) | 0.458 |
| - 20:53 1,4-xylene | 12.49 (1.37) | 12.00 (0.70) | 0.199 |
| - 27:90 Phenol | 16.26 (1.69) | 16.73 (2.84) | 0.459 |
| - 30:78 4-methylphenol | 19.59 (1.97) | 17.63 (2.13) | **0.004** |
| - 33:63 4-ethylphenol | 14.05 (0.60) | 14.02 (0.55) | 0.887 |
| - 38:39 1H-indole^&^ | 18.29 (1.28) | 17.54 (1.74) | 0.091 |
| - 41:12 3-methyl-1h-indole^&^ | 15.93 (2.02) | 14.45 (1.47) | **0.018** |
| **Alcohols;** **primary**^θ^**; secondary**^ρ^ | | | |
| - 17:26 Pentan-1-ol^θ^ | 12.22 (1.10) | 12.00 (0.74) | 0.479 |
| - 21:21 Hexan-1-ol^ρ^ | 12.55 (1.29) | 12.20 (0.72) | 0.332 |
| - 13:28 Butan-1-ol^θ^ | 13.06 (1.32) | 13.52 (1.69) | 0.300 |
| 25:10 Oct-1-en-3-ol^ρ^ | 13.06 (1.07) | 13.00 (1.07) | 0.862 |
| - 6:65 Ethanol^θ^ | 15.33 (0.49) | 15.93 (1.41) | **0.024** |
| - 9:54 Propan-1-ol^θ^ | 13.52 (1.34) | 15.18 (1.99) | **0.001** |
| **Organosulfur compounds** | | | |
| - 15:92 (Methyldisulfanyl) methane | 14.69 (2.53) | 13.59 (1.76) | 0.137 |
| - 25:22 (Methyltrisulfanyl) methane | 14.24 (2.07) | 13.34 (2.01) | 0.161 |
| **Monoterpenes; Terpenoids**^®^ | | | |
| - 22:55 Alpha-pinene^2^ | 13.29 (1.20) | 12.65 (0.79) | 0.073 |
| - 26:10 D-Limonene^3^ | 15.13 (1.86) | 14.25 (2.17) | 0.146 |
| - 26:91 Gamma-terpinene^4^ | 12.52 (1.33) | 12.29 (1.00) | 0.549 |
| - 28:98 Linalool^5^ | 11.87 (0.78) | 11.88 (0.67) | 0.989 |
| - 30:81 Cyclohexa-1,4-diene^®^ | 18.60 (0.99) | 18.44 (0.82) | 0.582 |
| **Ester** | | | |
| - 14:28 Propyl acetate | 12.55 (0.66) | 13.30 (1.51) | **0.014** |
| - 24:69 Butyl butanoate | 12.89 (1.04) | 13.57 (1.84) | 0.094 |
| - 17:93 Propyl propanoate | 11.85 (1.10) | 12.92 (0.49) | **0.001** |
| - 17:55 Ethyl butanoate | 12.64 (0.54) | 13.99 (1.92) | **<0.001** |
| **Nitrogenous compounds** | | | |
| - 23:86 Oxime-, methoxy-phenyl-^6^ | 15.54 (0.34) | 15.82 (0.75) | 0.059 |
| **Sesquiterpene; tricyclic**^π^ **or primary**^Ω^ |  |  |  |
| - 37:56 Alpha-Cubebene^7π^ | 12.39 (1.18) | 11.80 (0.74) | 0.088 |
| 39:51 β-Caryophyllene^8Ω^ | 14.21 (1.30) | 12.97 (1.32) | **0.004** |
| **Unclassified** | | | |
| - 24:31 (1S)-6,6-dimethyl-4-methylidenebicyclo [3.1.1] heptane | 13.91 (1.40) | 13.11 (1.24) | 0.065 |

**Notes:**

1. Student’s t-test
2. 2,6,6-trimethyl-bicyclo 3.1.1 hept-2-ene
3. (4R)-1-methyl-4-prop-1-en-2-ylcyclohexene
4. 1-methyl-4-propan-2-ylcyclohexa-1,4-diene
5. 3,7-dimethylocta-1,6-dien-3-ol
6. Methyl-ZN-hydroxy-benzene-carboximidate
7. (1R,5S,6R,7S,10R)-4,10-dimethyl-7-propan-2-yltricyclo [4.4.0.01,5] dec-3-ene_(alpha-Cubebene)
8. (1R,4E,9S)-4,11,11-trimethyl-8-methylidenebicyclo [7.2.0] undec-4-ene_(Caryophyllene)

Statistically-significant differences are shown in bold type

**STable 16: VOCS abundance at baseline and follow-up in ulcerative colitis**

| **Retention time and faecal volatile organic compound** | **Baseline vs follow-up (n=27 pairs)** | | | **Baseline vs remission in follow-up (n=10 pairs)** | | | **Baseline vs active disease in follow-up (n=17 pairs)** | | |
| --- | --- | --- | --- | --- | --- | --- | --- | --- | --- |
|  | **t stat** | **Higher in** | **P value^1^** | **T stat** | **Higher in** | **P value** | **T stat** | **Higher in** | **P value** |
| **Ketones or diketones*** | | | | | | | | | |
| - 10:50 Butan-2-one | -1.05 | FUP | 0.30 | -1.25 | FUP | 0.24 | -0.38 | FUP | 0.71 |
| - 10:13 Butane-2,3-dione* | -1.20 | FUP | 0.24 | -0.31 | FUP | 0.77 | -1.25 | FUP | 0.23 |
| - 13:73 Pentan-2-one | -0.77 | FUP | 0.45 | -0.44 | FUP | 0.67 | -0.62 | FUP | 0.54 |
| - 14:06 Pentane-2,3-dione | 2.08 | BL | 0.05 | 0.26 | BL | 0.80 | 2.50 | BL | **0.02** |
| - 17:98 Hexan-2-one | -1.07 | FUP | 0.30 | -1.22 | FUP | 0.25 | -0.45 | FUP | 0.66 |
| - 21:86 Heptan-2-one* | -0.91 | FUP | 0.37 | -1.89 | FUP | 0.09 | 0.13 | BL | 0.90 |
| - 25:56 Octan-2-one | -0.29 | FUP | 0.78 | -0.95 | FUP | 0.37 | 0.00 | FUP | 1.00 |
| - 29:00 Nonan-2-one | 0.62 | BL | 0.54 | -0.10 | FUP | 0.93 | 0.90 | BL | 0.38 |
| - 7:37 Propan-2-one (acetone) | 0.08 | BL | 0.93 | -0.01 | FUP | 0.99 | 0.13 | BL | 0.90 |
| - 16:84 2-Methylpent-1-en-3-one | -0.31 | FUP | 0.76 | -0.35 | FUP | 0.73 | -0.05 | FUP | 0.96 |
| - 13:08 3-Methylbut-3-en-2-one | -0.81 | FUP | 0.43 | -1.16 | FUP | 0.28 | 0.22 | BL | 0.83 |
| - 24:71 5-Methylheptan-2-one | -0.55 | FUP | 0.59 | -0.89 | FUP | 0.39 | 0.03 | BL | 0.97 |
| - 25:46 6-methylhept-5-en-2-one* | -1.23 | FUP | 0.23 | -1.00 | FUP | 0.34 | -0.73 | FUP | 0.48 |
| **Fatty Acids – short chain; saturated** | | | | | | | | | |
| - 12:43 Acetic acid | -0.80 | FUP | 0.43 | -1.16 | FUP | 0.28 | -0.06 | FUP | 0.96 |
| - 19:45 Butanoic acid | -0.12 | FUP | 0.91 | -0.08 | FUP | 0.94 | -0.09 | FUP | 0.93 |
| - 16:03 Propanoic acid | 0.05 | BL | 0.96 | -0.40 | FUP | 0.70 | 0.38 | BL | 0.71 |
| **Fatty Acids – branched-chain; saturated**^$^ | | | | | | | | | |
| - 18:15 2-Methylpropanoic acid | -0.09 | FUP | 0.93 | -0.18 | FUP | 0.86 | 0.03 | BL | 0.98 |
| - 21:38 3-Methylbutanoic acid^$^ | -0.58 | FUP | 0.56 | -0.29 | FUP | 0.78 | -0.51 | FUP | 0.62 |
| - 21:73 2-Methylbutanoic acid | -0.84 | FUP | 0.41 | -0.61 | FUP | 0.56 | -0.55 | FUP | 0.59 |
| - 25:25 4-Methylpentanoic acid | -0.16 | FUP | 0.87 | 0.31 | BL | 0.76 | -0.41 | FUP | 0.69 |
| **Fatty Acids – Medium-chain/ saturated** | | | | | | | | | |
| - 22:94 Pentanoic acid | 0.52 | BL | 0.61 | 0.80 | BL | 0.45 | 0.12 | BL | 0.91 |
| - 26:25 Hexanoic acid | 0.13 | BL | 0.90 | 0.05 | BL | 0.96 | 0.12 | BL | 0.91 |
| - 29:20 Hepatanoic acid | 0.87 | BL | 0.39 | 0.46 | BL | 0.66 | 0.72 | BL | 0.48 |
| **Monocarboxylic acid** | | | | | | | | | |
| - 31:48 Cyclohexanecarboxylic acid | -0.58 | FUP | 0.57 | -0.52 | FUP | 0.62 | -0.39 | FUP | 0.70 |
| **Aldehydes; methylate aldehydes**^α^**; alpha hydrogen**^β^**; aromatic aldehyde**^γ^ | | | | | | | | | |
| - 12:47 3-methylbutanal^α^ | -0.58 | FUP | 0.57 | 0.35 | BL | 0.73 | -1.15 | FUP | 0.27 |
| - 12:72 2-methylbutanal^α^ | 0.05 | BL | 0.96 | 0.12 | BL | 0.91 | -0.04 | FUP | 0.97 |
| - 18:21 Hexanal | -0.90 | FUP | 0.37 | -0.08 | FUP | 0.93 | -0.94 | FUP | 0.36 |
| - 22:15 Heptanal | -0.90 | FUP | 0.38 | -0.96 | FUP | 0.36 | -0.45 | FUP | 0.66 |
| - 23:39 3-methylsulfanylpropanal^β^ | -0.05 | FUP | 0.96 | -0.65 | FUP | 0.53 | 0.54 | BL | 0.60 |
| - 25:38 Benzaldehyde^γ^ | 1.71 | BL | 0.10 | 0.14 | BL | 0.89 | 2.36 | BL | **0.03** |
| - 25:88 Octanal | 1.39 | BL | 0.18 | -0.16 | FUP | 0.88 | 1.92 | BL | 0.07 |
| - 28:53 2-phenylacetaldehyde | 2.05 | BL | 0.05 | 0.66 | BL | 0.53 | 2.25 | BL | **0.04** |
| - 29:39 Nonanal | 1.18 | BL | 0.25 | -0.82 | FUP | 0.43 | 2.32 | BL | **0.03** |
| - 9:00 2-methylpropanal^α^ | 0.34 | BL | 0.74 | -0.46 | FUP | 0.66 | 0.85 | BL | 0.41 |
| **Aromatic compounds; aromatic hydrocarbon**^@^**; aromatic heterocyclic**^&^ | | | | | | | | | |
| - 20:23 Ethylbenzene^@^ | -1.93 | FUP | 0.06 | -1.98 | FUP | 0.08 | -0.92 | FUP | 0.37 |
| - 20:53 1,4-xylene | -0.49 | FUP | 0.63 | -0.86 | FUP | 0.41 | 0.12 | BL | 0.90. |
| - 27:90 Phenol | 2.89 | BL | **0.01** | 1.41 | BL | 0.19 | 2.56 | BL | **0.02** |
| - 30:78 4-methylphenol | -0.41 | FUP | 0.69 | -0.40 | FUP | 0.70 | -0.15 | FUP | 0.88 |
| - 33:63 4-ethylphenol | 1.38 | BL | 0.18 | -0.43 | FUP | 0.68 | 1.49 | BL | 0.16 |
| - 38:39 1H-indole^&^ | 1.18 | BL | 0.25 | 0.09 | BL | 0.93 | 1.73 | BL | 0.10 |
| - 41:12 3-methyl-1h-indole^&^ | 0.69 | BL | 0.50 | 0.08 | BL | 0.94 | 0.86 | BL | 0.40 |
| **Alcohols;** **primary**^θ^**; secondary**^ρ^ | | | | | | | | | |
| - 17:26 Pentan-1-ol^θ^ | -0.46 | FUP | 0.65 | 0.20 | BL | 0.85 | -0.68 | FUP | 0.51 |
| - 21:21 Hexan-1-ol^ρ^ | -0.93 | FUP | 0.36 | -0.17 | FUP | 0.87 | -0.93 | FUP | 0.36 |
| - 13:28 Butan-1-ol | -0.38 | FUP | 0.71 | 0.50 | BL | 0.63 | -0.78 | FUP | 0.45 |
| - 25:10 Oct-1-en-3-ol^ρ^ | 2.62 | BL | **0.01** | 1.58 | BL | 0.15 | 2.07 | BL | 0.05 |
| - 6:65 Ethanol^θ^ | 1.25 | BL | 0.22 | 1.00 | BL | 0.34 | 0.80 | BL | 0.44 |
| - 9:54 Propan-1-ol^θ^ | 0.64 | BL | 0.53 | 2.31 | BL | 0.05 | -0.84 | FUP | 0.41 |
| **Organosulfur compounds** | | | | | | | | | |
| - 15:92 (Methyldisulfanyl) methane | 0.13 | BL | 0.89 | -0.48 | FUP | 0.65 | 0.58 | BL | 0.57 |
| - 25:22 (Methyltrisulfanyl) methane | -0.50 | FUP | 0.62 | 0.13 | BL | 0.90 | -0.77 | FUP | 0.45 |
| **Monoterpenes; Terpenoids**^®^ | | | | | | | | | |
| - 22:55 Alpha-pinene^2^ | 0.63 | BL | 0.53 | -0.22 | FUP | 0.83 | 0.81 | BL | 0.43 |
| - 26:10 D-Limonene^3^ | -0.96 | FUP | 0.35 | -1.48 | FUP | 0.17 | -0.12 | FUP | 0.90 |
| - 26:91 Gamma-terpinene^4^ | -1.72 | FUP | 0.10 | -0.41 | FUP | 0.69 | -1.88 | FUP | 0.08 |
| - 28:98 Linalool^5^ | -1.22 | FUP | 0.23 | -0.97 | FUP | 0.36 | -0.76 | FUP | 0.46 |
| - 30:81 Cyclohexa-1,4-diene^®^ | 0.92 | BL | 0.36 | -1.32 | FUP | 0.22 | 1.71 | BL | 0.11 |
| **Ester** | | | | | | | | | |
| - 14:28 Propyl acetate | -0.55 | FUP | 0.59 | 0.82 | BL | 0.43 | -1.42 | BL | 0.17 |
| - 24:69 Butyl butanoate | 0.85 | BL | 0.40 | 1.46 | BL | 0.18 | 0.01 | BL | 0.99 |
| - 17:93 Propyl propanoate | 1.03 | BL | 0.31 | 2.38 | BL | 0.04 | -0.34 | FUP | 0.74 |
| - 17:55 Ethyl butanoate | 0.47 | BL | 0.64 | 1.45 | BL | 0.18 | -0.37 | FUP | 0.72 |
| **Nitrogenous compounds** | | | | | | | | | |
| 23.86 Oxime-, methoxy-phenyl-^6^ | 0.78 | BL | 0.44 | 0.15 | BL | 0.89 | 0.91 | BL | 0.38 |
| 24.31_(1S)-6,6-dimethyl-4-methylidenebicyclo [3.1.1] heptane | -0.88 | FUP | 0.39 | -1.43 | FUP | 0.19 | -0.01 | FUP | 0.99 |
| **Sesquiterpene; tricyclic**^π^ **or primary**^Ω^ | | | | | | | | | |
| - 37:56 Alpha-Cubebene^7π^ | -1.29 | FUP | 0.21 | -0.63 | FUP | 0.54 | -1.11 | FUP | 0.28 |
| - 39:51 β-Caryophyllene^8Ω^ | -0.84 | FUP | 0.41 | -1.35 | FUP | 0.21 | -0.07 | FUP | 0.94 |
| **Unclassified** | | | | | | | | | |
| 24.31_(1S)-6,6-dimethyl-4-methylidenebicyclo [3.1.1] heptane | -0.88 | FUP | 0.39 | -1.43 | FUP | 0.19 | -0.01 | FUP | 0.99 |

**Notes:**

1. A paired t-test
2. 2,6,6-trimethyl-bicyclo 3.1.1 hept-2-ene
3. (4R)-1-methyl-4-prop-1-en-2-ylcyclohexene
4. 1-methyl-4-propan-2-ylcyclohexa-1,4-diene
5. 3,7-dimethylocta-1,6-dien-3-ol
6. Methyl-ZN-hydroxy-benzene-carboximidate
7. (1R,5S,6R,7S,10R)-4,10-dimethyl-7-propan-2-yltricyclo [4.4.0.01,5] dec-3-ene_(alpha-Cubebene)
8. (1R,4E,9S)-4,11,11-trimethyl-8-methylidenebicyclo [7.2.0] undec-4-ene_(Caryophyllene)

Statistically-significant differences are shown in bold type

**STable 17: Faecal volatile organic compounds according to IBD sub-type**

| **Retention time and Faecal volatile organic compound** | **Crohn’s disease**  **N=78**  **Mean (SD)** | **Ulcerative colitis**  **N=38**  **Mean (SD)** | **P-Value^1^** |
| --- | --- | --- | --- |
| **Ketones or diketones*** | | | |
| - 10:50 Butan-2-one | 14.20 (1.44) | 13.61 (1.70) | 0.068 |
| - 10:13 Butane-2,3-dione* | 14.37 (1.18) | 13.94 (1.30) | 0.093 |
| - 13:73 Pentan-2-one | 14.37 (1.42) | 14.39 (1.28) | 0.983 |
| - 14: 06 Pentane-2,3-dione | 14.89 (1.56) | 14.48 (1.58) | 0.194 |
| - 17:98 Hexan-2-one | 13.50 (1.38) | 13.05 (1.16) | 0.071 |
| - 21:86 Heptan-2-one* | 14.38 (1.34) | 13.66 (1.51) | **0.015** |
| - 25:56 Octan-2-one | 13.38 (1.04) | 13.21 (1.06) | 0.412 |
| - 29:00 Nonan-2-one | 13.11 (1.20) | 12.61 (0.80) | **0.009** |
| - 7:37 Propan-2-one (acetone) | 14.48 (1.08) | 14.79 (2.25) | 0.430 |
| - 16:84 2-Methylpent-1-en-3-one | 12.46 (1.52) | 12.30 (1.49) | 0.590 |
| - 13:08 3-Methylbut-3-en-2-one | 14.06 (0.61) | 14.02 (0.61) | 0.737 |
| - 24:71 5-Methylheptan-2-one | 13.61 (1.47) | 13.00 (1.24) | **0.020** |
| - 25:46 6-methylhept-5-en-2-one* | 15.08 (1.56) | 13.99 (1.55) | **0.001** |
| **Fatty Acids– short-chain; saturated** | | | |
| - 12:43 Acetic acid | 16.85 (2.21) | 16.33 (2.24) | 0.246 |
| - 19:45 Butanoic acid | 19.54 (1.53) | 18.59 (2.49) | **0.036** |
| - 16:03 Propanoic acid | 17.66 (1.67) | 16.79 (2.27) | **0.039** |
| **Fatty Acids – branched chain; saturated**^$^ | | |  |
| - 18:15 2-Methylpropanoic acid | 17.00 (1.86) | 16.29 (2.29) | 0.102 |
| - 21:38 3-Methylbutanoic acid^$^ | 18.50 (1.65) | 17.52 (2.52) | **0.033** |
| - 21:73 2-Methylbutanoic acid | 18.16 (1.72) | 17.10 (2.36) | **0.017** |
| - 25:25 4-Methylpentanoic acid | 15.56 (2.16) | 15.02 (2.24) | 0.226 |
| **Fatty Acids-** **medium-chain/ saturated** | | | |
| - 22:94 Pentanoic acid | 18.61 (1.85) | 17.59 (2.40) | **0.025** |
| - 26:25 Hexanoic acid | 16.56 (2.21) | 16.00 (2.36) | 0.222 |
| - 29:20 Hepatanoic acid | 14.83 (1.11) | 14.83 (1.17) | 0.998 |
| **Mono Carboxylic acid** |  |  |  |
| - 31:48 Cyclohexanecarboxylic acid | 13.93 (1.66) | 13.57 (1.35) | 0.218 |
| **Aldehydes; methylate aldehydes**^α^**; alpha hydrogen**^β^**; aromatic aldehyde**^γ^ | | | |
| - 12:47 3-methylbutanal^α^ | 15.00 (1.87) | 15.75 (1.55) | **0.025** |
| - 12:72 2-methylbutanal^α^ | 14.81 (1.72 | 14.87 (1.97) | 0.875 |
| - 18:21 Hexanal | 13.26 (1.50) | 13.48 (1.67) | 0.490 |
| - 22:15 Heptanal | 13.21 (1.06) | 12.75 (1.11) | **0.037** |
| - 23:39 3-methylsulfanylpropanal^β^ | 13.11 (1.30) | 12.66 (1.36) | 0.095 |
| - 25:38 Benzaldehyde^γ^ | 15.75 (1.33) | 14.87 (1.52) | **0.003** |
| - 25:88 Octanal | 13.23 (1.40) | 12.91 (1.26) | 0.215 |
| - 28:53 2-phenylacetaldehyde | 16.54 (1.21) | 15.99 (1.78) | 0.090 |
| - 29:39 Nonanal | 12.79 (1.18) | 12.48 (1.79) | 0.147 |
| - 9:00 2-methylpropanal^α^ | 13.28 (0.95) | 13.38 (1.00) | 0.610 |
| **Aromatic compounds; aromatic hydrocarbon**^@^**; aromatic heterocyclic**^&^ | | | |
| - 20:23 Ethylbenzene^@^ | 12.11 (0.96) | 11.80 (0.71) | 0.051 |
| - 20:53 1,4-xylene | 12.20 (0.91) | 12.11 (0.89) | 0.587 |
| - 27:90 Phenol | 17.94 (2.59) | 16.60 (2.60) | **0.011** |
| - 30:78 4-methylphenol | 19.33 (1.92) | 18.14 (2.33) | **0.008** |
| - 33:63 4-ethylphenol | 14.06 (0.63) | 13.92 (0.24) | 0.096 |
| - 38:39 1H-indole^&^ | 18.50 (1.56) | 17.85 (1.65) | 0.047 |
| - 41:12 3-methyl-1h-indole^&^ | 14.98 (1.64) | 14.94 (1.64) | 0.915 |
| **Alcohols;** **primary**^θ^**; secondary**^ρ^ | | | |
| - 17:26 Pentan-1-ol^θ^ | 12.66 (1.55) | 12.05 (0.82) | **0.006** |
| - 21:21 Hexan-1-ol^ρ^ | 12.52 (1.36) | 12.33 (0.98) | 0.396 |
| - 13:28 Butan-1-ol^θ^ | 13.81 (1.67) | 13.34 (1.54) | 0.141 |
| - 25:10 Oct-1-en-3-ol^ρ^ | 12.86 (0.85) | 13.08 (1.03) | 0.259 |
| - 6:65 Ethanol^θ^ | 15.80 (0.68) | 15.86 (1.41) | 0.794 |
| - 9:54 Propan-1-ol^θ^ | 15.27 (1.76) | 14.81 (1.96) | 0.229 |
| **Organosulfur compounds** | | | |
| - 15:92 (Methyldisulfanyl) methane | 14.05 (1.88) | 13.56 (1.75) | 0.167 |
| - 25:22 (Methyltrisulfanyl) methane | 13.94 (1.82) | 13.31 (1.89) | 0.094 |
| **Monoterpenes: Terpenoids**^®^ | | | |
| - 22:55 Alpha-pinene^2^ | 12.93 (1.08) | 12.96 (1.10) | 0.901 |
| - 26:10 D-Limonene^3^ | 14.83 (1.56) | 14.49 (2.15) | 0.383 |
| - 26:91 Gamma-terpinene^4^ | 12.91 (1.84) | 12.10 (0.61) | **0.001** |
| - 28:98 Linalool^5^ | 12.12 (1.00) | 11.82 (0.67) | 0.063 |
| - 30:81 Cyclohexa-1,4-diene^®^ | 18.81 (1.08) | 18.56 (0.92) | 0.195 |
| **Ester** | | | |
| - 14:28 Propyl acetate | 13.01 (1.25) | 12.86 (1.03) | 0.482 |
| - 24:69 Butyl butanoate | 13.57 (1.47) | 13.28 (1.39) | 0.296 |
| - 17:93 Propyl propanoate | 12.38 (1.24) | 12.46 (1.18) | 0.739 |
| - 17:55 Ethyl butanoate | 13.42 (1.47) | 13.45 (1.56) | 0.911 |
| **Nitrogenous compounds** | | | |
| - 23:86 Oxime-, methoxy-phenyl-^6^_ | 15.69 (0.93) | 15.73 (0.72) | 0.772 |
| **Sesquiterpene; tricyclic**^π^ **or primary**^Ω^ | | | |
| - 37:56 Alpha-Cubebene^7π^ | 12.20 (1.18) | 11.89 (0.71) | 0.084 |
| 39:51 β-Caryophyllene^8Ω^ | 13.72 (1.58) | 13.18 (1.37) | 0.064 |
| **Unclassified** | | | |
| - 24:31 (1S)-6,6-dimethyl-4-methylidenebicyclo [3.1.1] heptane | 13.58 (1.58) | 13.24 (1.29) | 0.214 |

**Notes:**

1. Student’s t-test
2. 2,6,6-trimethyl-bicyclo 3.1.1 hept-2-ene
3. (4R)-1-methyl-4-prop-1-en-2-ylcyclohexene
4. 1-methyl-4-propan-2-ylcyclohexa-1,4-diene
5. 3,7-dimethylocta-1,6-dien-3-ol
6. Methyl-ZN-hydroxy-benzene-carboximidate
7. (1R,5S,6R,7S,10R)-4,10-dimethyl-7-propan-2-yltricyclo [4.4.0.01,5] dec-3-ene_(alpha-Cubebene)
8. (1R,4E,9S)-4,11,11-trimethyl-8-methylidenebicyclo [7.2.0] undec-4-ene_(Caryophyllen)

Statistically-significant differences are shown in bold type

**STable 18: Faecal volatile organic compounds according to small bowel verses large bowel disease**

| **Retention time and faecal volatile organic compound** | **CD (L1 only)**  **N=17**  **Mean (SD)** | **Colonic disease**  **(L2 + E1 + E2 + E3 + E4)**  **N=74**  **Mean (SD)** | **P-Value^1^** |
| --- | --- | --- | --- |
| **Ketones or diketones*** | | | |
| - 10:50 Butan-2-one | 14.85 (1.23) | 13.93 (1.66) | **0.014** |
| - 10:13 Butane-2,3-dione* | 14.35 (1.13) | 14.22 (1.28) | 0.687 |
| - 13:73 Pentan-2-one | 14.67 (1.49) | 14.53 (1.40) | 0.719 |
| - 14: 06 Pentane-2,3-dione | 15.37 (1.29) | 14.82 (1.70) | 0.145 |
| - 17:98 Hexan-2-one | 14.35 (1.63) | 13.41 (1.42) | **0.039** |
| - 21:86 Heptan-2-one* | 15.00 (1.12) | 13.93 (1.60) | **0.003** |
| - 25:56 Octan-2-one | 13.77 (1.28) | 13.31 (1.18) | 0.194 |
| - 29:00 Nonan-2-one | 13.71 (1.52) | 12.70 (0.93) | **0.017** |
| - 7:37 Propan-2-one (acetone) | 14.46 (1.13) | 14.70 (1.79) | 0.491 |
| - 16:84 2-Methylpent-1-en-3-one | 13.06 (1.55) | 12.17 (1.36) | **0.041** |
| - 13:08 3-Methylbut-3-en-2-one | 14.14 (0.64) | 14.02 (0.57) | 0.476 |
| - 24:71 5-Methylheptan-2-one | 14.39 (1.41) | 13.09 (1.29) | **0.002** |
| - 25:46 6-methylhept-5-en-2-one* | 15.79 (0.77) | 14.34 (1.59) | **<0.001** |
| **Fatty Acids – short chain; saturated** | | | |
| - 12:43 Acetic acid | 16.80 (2.05) | 16.73 (2.25) | 0.908 |
| - 19:45 Butanoic acid | 19.66 (1.03) | 18.97 (2.19) | 0.060 |
| - 16:03 Propanoic acid | 17.99 (1.24) | 17.07 (2.07) | **0.021** |
| **Fatty Acids – branched-chain; saturated**^$^ | | | |
| - 18:15 2-Methylpropanoic acid | 17.61 (1.14) | 16.66 (2.13) | **0.014** |
| - 21:38 3-Methylbutanoic acid^$^ | 18.71 (0.85) | 17.97 (2.26) | **0.030** |
| - 21:73 2-Methylbutanoic acid | 18.48 (0.97) | 17.52 (2.18) | **0.007** |
| - 25:25 4-Methylpentanoic acid | 15.93 (2.20) | 15.31 (2.19) | 0.305 |
| **Fatty Acids – medium-chain; saturated** | | | |
| - 22:94 Pentanoic acid | 19.03 (1.42) | 19.93 (2.22) | **0.015** |
| - 26:25 Hexanoic acid | 17.09 (2.21) | 16.29 (2.36) | 0.194 |
| - 29:20 Hepatanoic acid | 15.04 (1.24) | 14.96 (1.31) | 0.815 |
| **Monocarboxylic acid** | | | |
| - 31:48 Cyclo-hexane-carboxylic acid | 14.00 (1.56) | 13.77 (1.46) | 0.572 |
| **Aldehydes; methylate aldehydes**^α^**; alpha hydrogen**^β^**; aromatic aldehyde**^γ^ | | | |
| - 12:47 3-methylbutanal^α^ | 15.79 (1.18) | 15.32 (1.85) | 0.201 |
| - 12:72 2-methylbutanal^α^ | 15.46 (1.00) | 14.76 (1.81) | **0.035** |
| - 18:21 Hexanal | 13.66 (1.67) | 13.20 (1.62) | 0.315 |
| - 22:15 Heptanal | 13.16 (1.02) | 12.92 (1.13) | 0.395 |
| - 23:39 3-methylsulfanylpropanal^β^ | 13.72 (1.16) | 12.79 (1.29) | **0.007** |
| - 25:38 Benzaldehyde^γ^ | 16.16 (1.36) | 15.15 (1.58) | **0.014** |
| - 25:88 Octanal | 13.58 (1.57) | 12.98 (1.25) | 0.158 |
| - 28:53 2-phenylacetaldehyde | 17.07 (0.64) | 16.04 (1.63) | **<0.001** |
| - 29:39 Nonanal | 12.96 (1.32) | 12.46 (0.95) | 0.158 |
| - 9:00 2-methylpropanal^α^ | 13.46 (1.20) | 13.30 (0.92) | 0.619 |
| **Aromatic compounds; aromatic hydrocarbon**^@^**; aromatic heterocyclic**^&^ | | | |
| - 20:23 Ethylbenzene^@^ | 12.50 (1.10) | 11.98 (0.94) | 0.086 |
| - 20:53 1,4-xylene | 12.23 (0.85) | 12.18 (0.99) | 0.847 |
| - 27:90 Phenol | 17.86 (2.68) | 16.91 (2.54) | 0.195 |
| - 30:78 4-methylphenol | 20.12 (1.49) | 18.43 (2.21) | **0.001** |
| - 33:63 4-ethylphenol | 13.87 (0.05) | 14.02 (0.52) | **0.017** |
| - 38:39 1H-indole^&^ | 19.01 (1.37) | 17.92 (1.67) | **0.008** |
| - 41:12 3-methyl-1h-indole^&^ | 15.22 (1.81) | 14.87 (1.75) | 0.476 |
| **Alcohols;** **primary**^θ^**; secondary**^ρ^ | | | |
| - 17:26 Pentan-1-ol^θ^ | 13.30 (2.06) | 12.18 (1.00) | **0.041** |
| - 21:21 Hexan-1-ol^ρ^ | 12.24 (0.88) | 12.40 (1.15) | 0.531 |
| - 13:28 Butan-1-ol^θ^ | 13.69 (1.76) | 13.52 (1.62) | 0.711 |
| - 25:10 Oct-1-en-3-ol^ρ^ | 12.57 (0.45) | 13.00 (0.98) | **0.009** |
| - 6:65 Ethanol^θ^ | 15.74 (0.61) | 15.74 (1.10) | 0.984 |
| - 9:54 Propan-1-ol^θ^ | 15.15 (1.91) | 14.75 (1.91) | 0.444 |
| **Organosulfur compounds** | | | |
| - 15:92 (Methyldisulfanyl) methane | 14.41 (1.88) | 14.01 (2.01) | 0.442 |
| - 25:22 (Methyltrisulfanyl) methane | 14.13 (1.71) | 13.78 (2.04) | 0.473 |
| **Monoterpenes; Terpenoids**^®^ | | | |
| - 22:55 Alpha-pinene^2^ | 13.23 (1.40) | 12.78 (0.92) | 0.221 |
| - 26:10 D-Limonene^3^ | 15.91 (1.04) | 14.51 (1.99) | **<0.001** |
| - 26:91 Gamma-terpinene^4^ | 14.12 (2.75) | 12.38 (1.05) | **0.020** |
| - 28:98 Linalool^5^ | 12.11 (0.96) | 11.92 (0.81) | 0.460 |
| - 30:81 Cyclohexa-1,4-diene^®^ | 18.99 (1.20) | 18.60 (0.97) | 0.230 |
| **Ester** | | | |
| - 14:28 Propyl acetate | 13.07 (1.52) | 12.92 (1.20) | 0.711 |
| - 24:69 Butyl butanoate | 13.63 (1.54) | 13.34 (1.59) | 0.498 |
| - 17:93 Propyl propanoate | 12.41 (1.56) | 12.46 (1.22) | 0.904 |
| - 17:55 Ethyl butanoate | 13.25 (1.53) | 13.50 (1.64) | 0.554 |
| **Nitrogenous compounds** | | | |
| - 23:86 Oxime-, methoxy-phenyl-^6^ | 15.92 (1.17) | 15.70 (0.67) | 0.461 |
| **Sesquiterpene; tricyclic**^π^ **or primary**^Ω^ |  |  |  |
| - 37:56 Alpha-Cubebene^7π^ | 12.79 (1.75) | 12.06 (1.02) | 0.114 |
| - 39:51 β-Caryophyllene^8Ω^ | 14.75 (1.62) | 13.38 (1.46) | **0.004** |
| **Unclassified** | | | |
| - 24:31 (1S)-6,6-dimethyl-4-methylidenebicyclo [3.1.1] heptane | 14.80 (1.80) | 13.23 (1.24) | **0.003** |

**Notes:**

1. Student’s t-test
2. 2,6,6-trimethyl-bicyclo 3.1.1 hept-2-ene
3. (4R)-1-methyl-4-prop-1-en-2-ylcyclohexene
4. 1-methyl-4-propan-2-ylcyclohexa-1,4-diene
5. 3,7-dimethylocta-1,6-dien-3-ol
6. Methyl-ZN-hydroxy-benzene-carboximidate
7. (1R,5S,6R,7S,10R)-4,10-dimethyl-7-propan-2-yltricyclo [4.4.0.01,5] dec-3-ene_(alpha-Cubebene)
8. (1R,4E,9S)-4,11,11-trimethyl-8-methylidenebicyclo [7.2.0] undec-4-ene_(Caryophylle

Statistically-significant differences are shown in bold type

**STable 19: Summary of VOCs that differed significantly in abundance according to clinical parameters in Crohn’s disease and colitis**^1^

| **Volatile organic compound** | **Crohn’s disease** | | | | **UC and Colitis** | | | | **Crohn’s vs. UC** |
| --- | --- | --- | --- | --- | --- | --- | --- | --- | --- |
|  | **vs control** | **Severity**^2^ | **Distribution**^3^ | **Treatment**^4^ | **vs control in UC** | **Severity in colitis**^2^ | **Distribution in colitis**^5^ | **Treatment in UC**^4^ |  |
| **Ketones/Diketones^+^** | | | | | | | | | |
| - Butan-2-one | ↓** | - | - | - | ↓* | - | - | - | - |
| - Butane-2,3-dione**^+^** | - | - | - | - | ↓* | (**) | - | - | - |
| - Pentan-2-one | - | - | - | - | - | - | - | - | - |
| - Pentane-2,3-dione | - | - | ↓*L3 |  | ↓* | - | - | - | - |
| - Hexan-2-one | - | - | ↓*L3 | - | ↓* | - | - | - | ↑* |
| - Heptan-2-one**^+^** | ↓* | - | - | - | ↓** | ↓* | - | - | - |
| - Octan-2-one | - | - | - | - | ↓** | - | - | - | - |
| - Nonan-2-one | - | - | - | - | ↓* | - | - | - | ↑** |
| - Propan-2-one (acetone) | - | - | - | - | - | ↑* | - | - | - |
| - 2-Methylpent-1-en-3-one | - | - | - | - | - | ↓* | - | - | - |
| - 3-Methylbut-3-en-2-one | - | - | - | - | - | - | ↓* | - | - |
| - 5-Methylheptan-2-one | - | - | ↑*L1 | - | ↓** | - | - | - | ↑* |
| - 6-methylhept-5-en-2-one**^+^** | - | (*) | - | - | ↓** | ↓*** | ↓* | - | ↑** |
| **Fatty Acids – short-chain; saturated** | | | | | | | | | |
| - Acetic acid | - | - | - | - | - | ↓** | - |  | - |
| - Butanoic acid | - | ↓* | - | - | - | ↓*** | - |  | ↑* |
| - Propanoic acid | - | - | - | - | - | ↓*** | - |  | ↑* |
| **Fatty Acids – branched-chain; saturated** | | | | | | | | | |
| - 2-Methylpropanoic acid | - | (*) | - | - | - | ↓** | - | - | - |
| - 3-Methylbutanoic acid^+^ | - | ↓** | - | - | - | ↓*** | - |  | ↑* |
| - 2-Methylbutanoic acid | - | ↓** | - | - | ↓* | ↓** | - | - | ↑* |
| **Fatty Acids – medium-chain; saturated** | | | | | | | | |  |
| - Pentanoic acid | - | ↓** | - | - | ↓* | ↓*** | - | - | ↑* |
| - Hexanoic acid | ↓* | ↓* | - | - | - | - | - | - | - |
| **Monocarboxylic acid** | | | | | | | | | |
| - Cyclohexanecarboxylic acid | - | - | - | - | - | - | ↑* | - | - |
| **Aldehydes; alpha-hydrogen^+1^; aromatic aldehyde^+2^; methylate aldehydes**^+3^ | | | | | | | | |  |
| - 3-methylbutanal^+3^ | - | - | - | - | - | - | - | - | ↓* |
| - Hexanal | - | - | - | ↓* | - | - | - | - | - |
| - Heptanal | - | - | - | - | - | - | - | - | ↑* |
| - 3-methyl-sulfanyl-propanal^+1^ | ↓*** | - | - | - | ↓** | ↓* | - | - | - |
| - Benzaldehyde^+2^ | - | - | - | - | - | ↓* | - | - | ↑** |
| - Octanal | ↓* | - | - | - | ↓* | ↓** | - | - | - |
| - 2-phenylacetaldehyde | ↓** | - | - | - | - | ↓** | - | - | - |
| - Nonanal | - | - | - | - | ↓* | - | - | - | - |
| **Aromatic compounds; aromatic hydrocarbon**^+^ | | | | | | | | |  |
| - Ethylbenzene^+^ | - | - | - | - | ↓** | - | - | - | - |
| - Phenol | ↑*** | - | - | ↓* | - | ↓* | - | ↓* | ↑* |
| - 4-methylphenol | - | - | - | - | ↓*** | ↓* | ↓** | - | ↑** |
| - 4-ethylphenol | - | - | - | ↑* | - | - | - | - | ↑* |
| - 3-methyl-1h-indole^+^ | - | - | - | - | - | - | ↓* | - | - |
| **Alcohols – primary; secondary**^+^ | | | | | | | | |  |
| - Pentan-1-ol | - | - | - | - | ↓** | - | - | - | ↑** |
| - Hexan-1-ol^+^ | - | - | - | - | - | - | - | - | - |
| - Oct-1-en-3-ol^+^ | ↑** | - | - | - | ↑* | - | - | ↓* | - |
| - Ethanol | ↑** | - | - | ↓* | - | - | ↑* | - | - |
| - Propan-1-ol | ↑*** | - | - | ↓*** | - | - | ↑** | - | - |
| **Organosulfur compounds** | | | | | | | | |  |
| - (Methyldisulfanyl) methane | ↓* | - | - | - | ↓* | - | - | - | - |
| - (Methyltrisulfanyl) methane | - | - | - | - | - | - | - | - | - |
| **Monoterpenes/ Terpenoids** | | | | | | | | |  |
| - D-Limonene | ↓*** | ↓* | ↑**L1 | - | ↓** | - | - | - | - |
| - Gamma-terpinene | - | - | ↑**L1 | - | ↓** | - | - | - | ↑** |
| **Ester** | | | | | | | | |  |
| - Propyl acetate | - | - | - | - | - | - | ↑* | - | - |
| - Propyl propanoate | - | - | - | - | - | - | ↑** | - | - |
| - Ethyl butanoate | - | - | - | - | - | - | ↑*** | - | - |
| **Nitrogenous compounds** | | | | | | | | |  |
| - Methyl-ZN-hydroxy-benzene-carboximidate | - | ↑* | - | - | - | - | - | - | - |
| **Sesquiterpene/ primary^+1^/ tricyclic^+2^** | | | | | | | | |  |
| - Alpha-Cubebene^+2^ | ↓* | - | ↓*L3 | - | - | ↓* | - | - | - |
| - β-Caryophyllene^+1^ | ↓* | - | ↑**L1 | - | ↓** | ↓** | ↓** | - | - |
| **Unclassified** | | | | | | | | | |
| - (1S)-6,6-dimethyl-4-methylidenebicyclo [3.1.1] heptane | - | - | ↑**L1 | - | ↓* | - | -- | - | - |

**Notes:**

1. * P<0.05; ** P<0.01; *** P<0.001
2. For severity assessed by disease activity score, (*) although statistically significant, there was no consistent trend according to severity categories (see STable 8 for CD and STable 14 for colitis)
3. L1 - distal 1/3 ileum with or without limited cecal disease; L2 – colonic disease; L3- ileocolonic disease
4. Baseline vs. follow-up
5. Extensive colitis (E3+E4) vs. distal inflammation (E1+E2); ↑ means greater in extensive disease

**STable 20: Faecal VOCS that were lower in abundance in IBD cases and matched controls at baseline grouped by compound type**

| **Compound** | **Faecal VOCs** | **Lower in abundance in this study** | **Source(s) for specific VOCs and evidence from other studies** |
| --- | --- | --- | --- |
| **Ketones** | Butan-2-one | CD and colitis | Arises from carbohydrate and fatty acid metabolism (1) and decarboxylation of fatty acid derives (2,3) |
|  | 3-Methylbut-3-en-2-one | CD only | Arises from fatty acid and carbohydrate metabolism. Produced by bacteria and fungi (4,5) |
|  | Octan-2-one | CD only | Produced by many species of bacteria and fungi (1) |
|  | Butane-2,3-dione | Colitis only | Derived from pyruvate fermentation under anaerobic conditions (6, 7). Influences the growth of certain bacteria and yeast (12, 13) |
|  | heptan-2-one | Colitis only | Abundant in cheddar cheese and potatoes (1). May play a part in inhibiting enteric *Escherichia coli* (8) |
|  | 6-methylhept-5-en-2-one | Colitis only | Abundant in carrots (4) |
|  | 5-Methylheptan-2-one | Colitis only | - |
|  | Hexan-2-one | Colitis only | Produced by bacteria like *Klebsiella pneumoniae* (9, 10) |
|  | Nonan-2-one | Colitis only | Produced by bacteria like *Klebsiella pneumoniae* and *E. coli* (9,10, 11) |
| **Aldehydes** | Octanal | CD and colitis | Produced by lipid peroxidation and oxidative stress during inflammatory processes (12, 13). GIT damage and mucosal ulceration in IBD |
|  | 2-phenylacetaldehyde | CD and colitis |  |
|  | 3-methyl-sulfanyl-propanal | CD and colitis |  |
|  | Nonanal | Colitis only | Found in potato tubers (1). Results from the reduction of the carboxy group of nonanoic acid in humans and plants. Metabolite detected in cancer metabolism (14) |
| **Saturated medium-chain fatty acids** | Hexanoic acid | CD only | Found in or produced by [*Saccharomyces cerevisiae*](https://pubchem.ncbi.nlm.nih.gov/taxonomy/4932) (15) |
|  | Pentanoic acid | Colitis only | Produced by the fermentation of carbohydrates in the gut, particularly *Bacteroides* (1, 16) |
|  | 2-Methylbutanoic acid | Colitis only | Arises from intestinal microbial action on proteins by the respective branched amino acid (1) |
| **Aromatics: low abundance of some aromatic compounds, including 4-methylphenol, in IBD** | 4-methylphenol | Colitis only | - |
|  | Ethylbenzene | Colitis only | May arise from air pollution (1) |
|  | 4-ethylphenol | Colitis only |  |
| **Terpenoids** | D-Limonene | CD and colitis | Present in many plants and is abundant in the peel of citrus fruits such as oranges (17). May have beneficial effects on colon lung cancer inducing apoptosis via a mechanism involving autophagy. Antioxidant and anti-inflammatory properties in a UC rat model (18,19) |
|  | Gamma-terpinene | Colitis only |  |
| **Sesquiterpene** | β-Caryophyllene | CD and colitis | Anti-inflammatory; possible therapy for IBD (18, 20) |
|  | Alpha-Cubebene | CD only | Anti-inflammatory; possible therapy for IBD (18,20) |
| **Organosulfur compounds** | (Methyldisulfanyl) methane | CD and colitis | Mainly produced by bacteria, e.g., pseudomonas aeruginosa and streptococcus pneumoniae (20, 21).  Produced by methylation of hydrogen sulfide as a detoxification mechanism by mucosal S-thiol methyltransferase (22).  Highly toxic and may induce inflammation (21). |
|  | (Methyltrisulfanyl) methane | Colitis only | Potential marker of *Pseudomonas aeruginosa* (20) |
| **Unclassified** | (1S)-6,6-dimethyl-4-methylidenebicyclo [3.1.1] heptane | Colitis only |  |

CD = Crohn’s disease; colitis = ulcerative colitis and inflammatory bowel disease unclassified

**References for STable 20:**

1. Garner CE, Smith S, de Lacy Costello B et al. Volatile organic compounds from feces and their potential for diagnosis of gastrointestinal disease. The FASEB Journal 2007;21:1675-88.
2. Monasta L, Pierobon C, Princivalle A et al. Inflammatory bowel disease and patterns of volatile organic compounds in the exhaled breath of children: A case-control study using Ion Molecule Reaction-Mass Spectrometry. PloS One 2017;12:e0184118.
3. Schulz S, Dickschat JS. Bacterial volatiles: the smell of small organisms. Nat Prod Rep 2007;24:814-42.
4. Kjeldsen F, Christensen LP, Edelenbos M. Quantitative analysis of aroma compounds in carrot (Daucus carota L.) cultivars by capillary gas chromatography using large-volume injection technique. J Agric Food Chem 2001;49:4342-8.
5. Decombaz J, Arnaud MJ, Milon H et al. Energy metabolism of medium-chain triglycerides versus carbohydrates during exercise. Eur J Appl Physiol Occup Physiol 1983;52:9-14.
6. Ryu CM, Farag MA, Hu CH et al. Bacterial volatiles promote growth in Arabidopsis. Proc Nat Acad Sci USA 2003;100:4927-32.
7. Ott A, Germond JE, Chaintreau A. Vicinal diketone formation in yogurt: 13C precursors and effect of branched-chain amino acids. J Agric Food Chem 2000;48:724-31.
8. Melkina OE, Khmel IA, Plyuta VA, Koksharova OA, Zavilgelsky GB. Ketones 2-heptanone, 2-nonanone, and 2-undecanone inhibit DnaK-dependent refolding of heat-inactivated bacterial luciferases in Escherichia coli cells lacking small chaperon IbpB. Appl Microbiol Biotechnol 2017;101:5765–5771.
9. Rees CA, Smolinska A, Hill JE. Volatile metabolic diversity of Klebsiella pneumoniae in nutrient-replete conditions. Metabolomics 2017;13(2):18.
10. Rees CA, Burklund A, Stefanuto P-H, Schwartzman JD, Hill JE. Comprehensive volatile metabolic fingerprinting of bacterial and fungal pathogen groups. J Breath Res 2018;12:026001.
11. Chen J, Tang J, Shi H, Tang C, Zhang R. Characteristics of volatile organic compounds produced from five pathogenic bacteria by headspace-solid phase micro-extraction/gas chromatography-mass spectrometry. J Basic Microbiol 2017;57,228–237.
12. Fritz KS, Petersen DR. An overview of the chemistry and biology of reactive aldehydes. Free Radic Biol Med 2013;59:85-91.
13. Rezaie A, Parker RD, Abdollahi M. Oxidative stress and pathogenesis of inflammatory bowel disease: an epiphenomenon or the cause? Dig Dis Sci 2007;52:2015-2021.
14. National Center for Biotechnology Information (2023). PubChem Compound Summary for CID 31289, Nonanal. Retrieved January 2, 2023 from <https://pubchem.ncbi.nlm.nih.gov/compound/Nonanal>.
15. National Center for Biotechnology Information (2023). PubChem Compound Summary for CID 8892, Hexanoic acid. Retrieved January 2, 2023 from <https://pubchem.ncbi.nlm.nih.gov/compound/Hexanoic-acid>.
16. Backhed F, Ley RE, Sonnenburg JL, Peterson DA, Gordon JI. Host-bacterial mutualism in the human intestine. Science 2005;307:1915-20.
17. Bakkali F, Averbeck S, Averbeck D, Idaomar M. Biological effects of essential oils–a review. Food Chem Toxicol 2008;46:446-75.
18. Yu X, Lin H, Wang Y et al. D-limonene exhibits antitumor activity by inducing autophagy and apoptosis in lung cancer. Onco Targets Ther 2018;11:1833-1847.
19. Yu L, Yan J, Sun Z. D-limonene exhibits anti-inflammatory and antioxidant properties in an ulcerative colitis rat model via regulation of iNOS, COX-2, PGE2 and ERK signaling pathways. Mol Med Rep 2017;15:2339-2346.
20. Bos LD, Sterk PJ, Schultz MJ. Volatile metabolites of pathogens: a systematic review. *PLoS Pathog*. 2013;9:e1003311.
21. Yoshimura M, Nakano Y, Yamashita Y, Oho T, Saito T, Koga T. Formation of methyl mercaptan froml-methionine by Porphyromonas gingivalis. Infect Immun 2000;68:6912-6916.
22. Roediger WE, Babidge WJ. Thiol methyltransferase activity in inflammatory bowel disease. Gut 2000;47:206-10.

1. Levine A, Griffiths A, Markowitz J et al. Pediatric modification of the Montreal classification for inflammatory bowel disease: the Paris classification. Inflamm Bowel Dis 2011;17:1314-21. [↑](#footnote-ref-1)
2. Ruemmele FM, Hyams JS, Otley A et al. Outcome measures for clinical trials in paediatric IBD: an evidence-based, expert-driven practical statement paper of the paediatric ECCO committee. Gut 2015;64:438-46. [↑](#footnote-ref-2)
3. Reade S, Mayor A, Aggio R et al. Optimisation of sample preparation for direct SPME-GC-MS analysis of murine and human faecal volatile organic compounds for metabolomic studies. J Anal Bioanal Tech 2014;5:184. [↑](#footnote-ref-3)
4. Reade S, Williams JM, Aggio R et al. Potential role of faecal volatile organic compounds as biomarkers of chemically induced intestinal inflammation in mice. FASEB J 2019;33:3129-36. [↑](#footnote-ref-4)
5. Bijlsma S, Bobeldijk I, Verheij ER et al. Large-scale human metabolomics studies: a strategy for data (pre-) processing and validation. Anal Chem 2006;78:567-74.

   ^6^ Aggio R, Villas− Bôas SG, Ruggiero K. Metab: an R package for high-throughput analysis of metabolomics data generated by GC-MS. Bioinformatics 2011;27:2316-8. [↑](#footnote-ref-5)
6. Ruemmele FM, Hyams JS, Otley A, et al. Outcome measures for clinical trials in paediatric IBD: an evidence-based, expert-driven practical statement paper of the paediatric ECCO committee. Gut 2015;64:438-46 [↑](#footnote-ref-6)
7. Levine A, Griffiths A, Markowitz J, et al. Pediatric modification of the Montreal classification for inflammatory bowel disease: the Paris classification. Inflamm Bowel Dis 2011;17:1314-21. [↑](#footnote-ref-7)
